# *Bifidobacterium* inhibits the progression of colorectal tumorigenesis in mice through fatty acid isomerization and gut microbiota modulation

**DOI:** 10.1080/19490976.2025.2464945

**Published:** 2025-02-09

**Authors:** Yang Chen, Huiting Fang, Haiqin Chen, Xiaoming Liu, Jianxin Zhao, Catherine Stanton, R. Paul Ross, Wei Chen, Bo Yang

**Affiliations:** a State Key Laboratory of Food Science and Resources, Jiangnan University, Wuxi, Jiangsu, China; b School of Food Science and Technology, Jiangnan University, Wuxi, Jiangsu, China; c International Joint Research Laboratory for Maternal-Infant Microbiota and Health, Jiangnan University, Wuxi, Jiangsu, China; d Teagasc Food Research Centre, Moorepark, Fermoy, Ireland; e APC Microbiome Ireland, University College Cork, Cork, Ireland

**Keywords:** Bifidobacterium, colorectal cancer, conjugated linoleic acid, PPAR-γ, Odoribacter splanchnicus, butyric acid, intestinal barrier

## Abstract

Colorectal cancer (CRC) represents the third most common cancer worldwide. Consequently, there is an urgent need to identify novel preventive and therapeutic strategies for CRC. This study aimed to screen for beneficial bacteria that have a preventive effect on CRC and to elucidate the potential mechanisms. Initially, we compared gut bacteria and bacterial metabolites of healthy volunteers and CRC patients, which demonstrated that intestinal conjugated linoleic acid (CLA), butyric acid, and *Bifidobacterium* in CRC patients were significantly lower than those in healthy volunteers, and these indicators were significantly negatively correlated with CRC. Next, spontaneous CRC mouse model were conducted to explore the effect of supplemental CLA-producing *Bifidobacterium* on CRC. Supplementation of mice with CLA-producing *Bifidobacterium breve* CCFM683 and *B. pseudocatenulatum* MY40C significantly prevented CRC. Moreover, molecular approaches demonstrated that CLA and the CLA-producing gene, *bbi*, were the key metabolites and genes for CCFM683 to prevent CRC. Inhibitor intervention results showed that PPAR-γ was the key receptor for preventing CRC. CCFM683 inhibited the NF-κB signaling pathway, up-regulated MUC2, Claudin-1, and ZO-1, and promoted tumor cell apoptosis via the CLA-PPAR-γ axis. Additionally, fecal microbiota transplantation (FMT) and metagenomic analysis showed that CCFM683 up-regulated *Odoribacter splanchnicus* through CLA production, which then prevented CRC by producing butyric acid, up-regulating TJ proteins, regulating cytokines, and regulating gut microbiota. These results will contribute to the clinical trials of *Bifidobacterium* and the theoretical research and development of CRC dietary products.

## 1. Introduction

Colorectal cancer (CRC) is a malignant tumor of the colon and rectum, ranking as the third most common cancer globally and the second in terms of mortality. In 2020, the global death toll exceeded half a million, with over 1.5 million new confirmed cases.^1^ Initial stages of CRC are asymptomatic, while intermediate stages may present symptoms such as diarrhea, abdominal pain, and bloody stools. Advanced stages may manifest systemic symptoms like anemia and dramatic weight loss.^2^ Environmental and genetic factors such as lack of exercise, obesity, alcohol consumption, smoking, unhealthy diet, and family history of colorectal polyps increase the risk of CRC.^3^ Additionally, dysbiosis of the gut microbiota is closely associated with CRC onset.^4^ Influenced by factors such as gut microbiota, environment, and genetics, certain normal epithelial cells in the colon of CRC patients may transform into intermediate adenomas or polyps, ultimately progressing to advanced adenocarcinoma. Therefore, prevention, treatment, and prognosis of CRC require attention.

Although there has been some progress in the diagnosis and early detection of CRC in recent years, most newly diagnosed CRC patients are diagnosed at advanced stages. Traditional therapies such as chemotherapy and radiotherapy cannot differentiate between cancerous and normal cells, inducing apoptosis in surrounding normal cells during treatment. Immunotherapy is merely a supplementary treatment and does not cure CRC. Current treatments have negative side-effects and high costs.^5^ In order to avoid these problems in the diagnosis and treatment of CRC in the middle and late stages, the prevention of CRC is very important. Therefore, seeking strategies to prevent CRC with minimal side effects is a current research hotspot. Probiotics can produce bacteriocins, compete for adhesion sites with harmful microbes, produce active metabolites, improve the intestinal epithelial barrier, and regulate intestinal immunity, all of which can help prevent CRC.^6^

*Bifidobacterium*, an important probiotic genus, has been shown to prevent CRC in various ways. Dietary supplementation with *B. bifidum* can promote apoptosis of colonic epithelial cells in CRC mice.^7^ Moreover, orally administered *B. longum* subsp. *infantis* can up-regulate the expression of Occludin and Claudin-1, and inhibit the activation of Caco-2 cells.^8^ Apart from regulating apoptosis and mechanical barriers, *Bifidobacterium* can prevent CRC by regulating intestinal immunity. For instance, orally administered *B. longum* subsp. *infantis* can significantly increase Treg cells in the colon of CRC rats, down-regulate IL-6, IL-1β, and TNF-α, and prevent CRC.^9^ Furthermore, regulation of the gut microbiota is a crucial way for *Bifidobacterium* to prevent CRC. For example, orally administered *B. longum* in CRC mice significantly up-regulated *Odoribacter* and *Lactobacillus*, improving the dysbiotic gut microbiota.^10^ However, the key metabolites, key receptors, key signaling pathways, and key gut microorganisms in preventing CRC are not clear, which hinders the development of *Bifidobacterium* dietary supplements.

Based on the results from clinical research, this study screened for beneficial bacteria that had a mitigating effect on CRC. Then the potential mechanisms for CRC prevention from aspects of key metabolite, key receptors, key signaling pathways, key microbes, and intestinal barrier were further analyzed, which will promote the clinical research of beneficial bacteria and the development of dietary supplements.

## 2. Materials and methods

### 2.1. Clinical sample collection

Fecal and serum samples of 40 healthy volunteers and 40 CRC patients from Wuxi Second People’s Hospital were selected for physical examination (Table S1). Inclusion criteria of the volunteers: 1. No history of previous tumors; 2. Age between 18 and 65 years; 3. Male or non-pregnant, non-lactating female; 4. No prior radiotherapy or chemotherapy before enrollment; 5. Able to provide sufficient and qualified blood and faeces samples; 6. Voluntary signature of informed consent. For the CRC group: 7. Preoperative colonoscopy pathology diagnosis of colorectal adenocarcinoma or high-grade adenoma. For the healthy group: 8. healthy individuals with no significant findings in colonoscopy pathology. 9. Other confirmed tumors unrelated to CRC, including gastric cancer, liver cancer, esophageal cancer, biliary cancer, pancreatic cancer, as well as common non-digestive system tumors such as lung cancer, breast cancer, cervical cancer, and thyroid cancer. Exclusion criteria of the volunteers: 1. Plasma or faeces contamination in the laboratory; 2. Participation in another clinical trial within 30 days before enrollment; 3. Pregnant or lactating women; 4. History of tumors and corresponding tumor treatments; 5. Blood transfusion within 30 days before blood collection; 6. History of organ, bone marrow, or stem cell transplantation; 7. Health conditions unsuitable for blood collection; 8. Incomplete clinical data (including tumor volume, pathological classification, site of occurrence, vascular invasion, age, sex, depth of invasion, degree of differentiation, alcohol history, smoking history, etc.). The Clinical Research Ethics Committee of Wuxi Second People’s Hospital approved the study protocol (2022Y-219). Fecal and serum samples of volunteers were collected during the physical examination and stored at –80°C.

### 2.2. Bacterial culture

*B. breve* CCFM683, *B. longum* CCFM681, *B. pseudocatenulatum* MY40C, and *Lactobacillus plantarum* ST-III used in this study were preserved in the Culture Collection of Food Microorganisms (CCFM), Jiangnan University. The linoleate isomerase knockout mutant (CCFM683Δ*bbi*), corresponding complemented mutants (CCFM683Δ*bbi:bbi*), and recombinant *L. plantarum* ST-III (ST-III/pNZ44-*bbi*) expressing linoleate isomerase were previously constructed.^11^ CCFM683, CCFM681, MY40C, and ST-III were cultured as previously described.^12^ *O. splanchnicus* (BNCC359789 = ATCC29572) was purchased from BeNa Culture Collection, Henan, China. The cultivation of *O. splanchnicus* was carried out according to the previously described method.^13^

### 2.3. Design of animal experiments

Three or five weeks old, male C57BL/6J Apc^Min+^ mice were supplied from the Institute of Model Zoology, Nanjing University (Nanjing, Jiangsu, China). The experiment program was approved by the Experimental Animal Ethics Committee of Jiangnan University (JN.No20190915c0241220[211],JN.No20220415c0480710[123],JN.No20220615c0180930[292],JN.No20211015c0400110[385],JN.No20220530c0800708[165],JN.No20220530c0400830[166]). The animal trials were divided into six batches.

The first batch of the animal trial was designed according to Figure 2a. Forty Apc^Min+^ mice were divided into five groups (*n* = 8 for each group) and fed with sterile water and standard chow. Briefly, the mice in the PBS group were gavaged with 0.2 mL/day PBS. Approximately 10^9^ CFU/day of CCFM683, MY40C, or CCFM681 were given to each mouse. Fresh suspension of CCFM683, MY40C, or CCFM681 was centrifuged at 3,000 g and 4℃ for 15 min daily and diluted with PBS to 5×10^9^ CFU/mL. The mice in the CLA group were administered 20 mg/day CLA (Nu-Chek-Prep, Elysian, MN, USA).

The second batch of the animal trial was designed according to Figure 3a. Thirty-two Apc^Min+^ mice were divided into four groups (*n* = 8 for each group) and fed with sterile water and standard chow. The experimental treatments for the mice in the PBS and *B. breve* groups were the same as those in Figure 2a. Fresh feces from the CCFM683 and CCFM683Δ*bbi* group of mice were collected in a precooled enzyme-free cryopreservation tube. The bacterial solution for fecal microbiota transplantation (FMT) was then prepared according to the previous method.^14^

The third batch of the animal test was designed according to Figure 4a. Twenty-four Apc^Min+^ mice were divided into three groups (*n* = 8 for each group) and fed with sterile water and standard chow. The experimental treatments for the mice in the PBS and *L. plantarum* groups were the same as those in Figure 2a.

**Figure 5.**
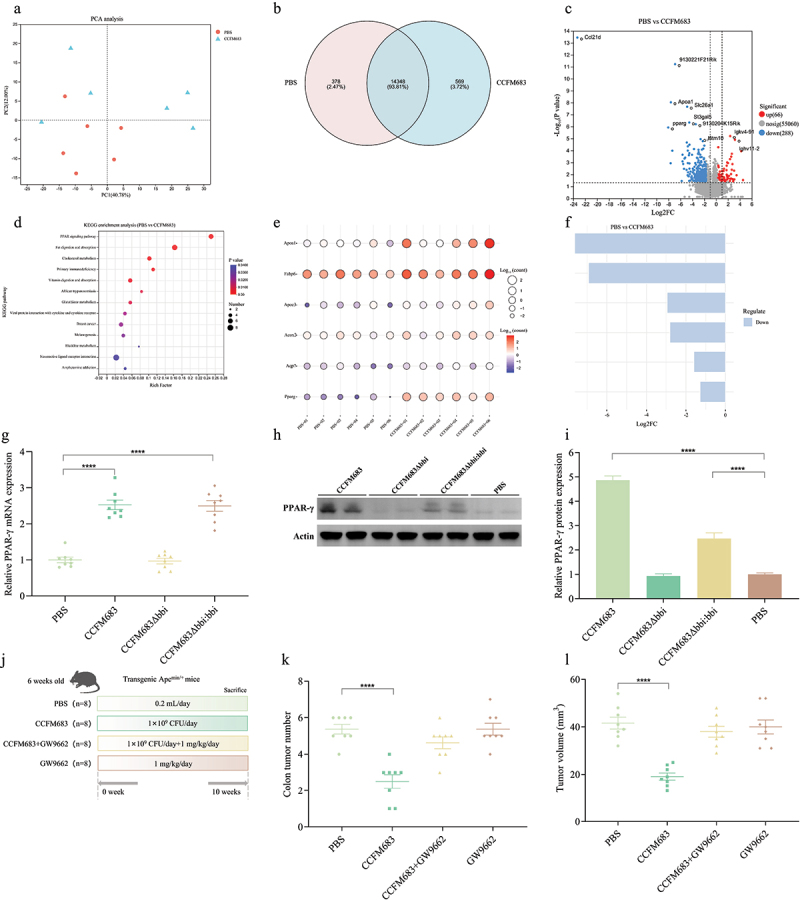
Analysis of key receptor of CCFM683 in relieving CRC.

The fourth batch of the animal trial was designed according to Figure 5j. In total, 32 Apc^Min+^ mice were divided into four groups (*n* = 8 for each group) and fed with sterile water and standard chow. The experimental treatments for the mice in the PBS and CCFM683 groups were the same as those in Figure 2a. In addition to the daily supplementation of 10^9^ CFU CCFM683, mice in the CCFM683+GW966 group were intraperitoneally injected with 1 mg/kg/day PPAR-γ inhibitor GW966 (Sigma Aldrich Trading Co., Ltd., Shanghai, China). The mice in the GW966 group were intraperitoneally injected with 1 mg/kg/day PPAR-γ inhibitor GW966 without CCFM683 administration.

(a) Between-sample diversity and clustering shown by PCA of the transcriptomes. (b) Venn diagram between PBS and CCFM683 groups. (c) Differentially expressed genes (log2FC≥|1| and adjust p-value<0.05) between PBS and CCFM683 groups, shown by volcano plot. (d) KEGG pathway enrichment analysis of the most significantly changed pathways. The size of the dot indicates the number of differently expressed genes in the signaling pathway. (e) Bubble diagram of differentially expressed genes in PPAR signaling pathway among PBS and CCFM683 treatments (log2FC≥|1| and adjust p-value <0.05). (f) Deviation diagram of differentially expressed genes in PPAR signaling pathway among PBS and CCFM683 treatments (log2FC≥|1| and adjust p-value <0.05). (g) The relative expression of mRNA PPAR-γ in the colon. (h-i) Relative PPAR-γ protein expression in the colon. (j) Experimental design for CCFM683, CCFM683+GW9662, and GW9662 treatments for an Apc^Min+^ mouse model. (k) The colon tumor number. (i) The colon tumor volume. (m) HE staining for mice colons, scale bars, 50 μm. (n) The percent of high-grade dysplastic adenocarcinoma of the colon. (o) Immunohistochemical staining for Ki67 and PCNA in the colon, scale bars, 50 μm. The mean optical density values of Ki67 (p) and PCNA (q) in the colon. (r) The concentration of PPAR-γ in the serum of healthy volunteers and CRC patients. The interdependent quantitative relationships between the concentration of PPAR-γ in serum and CLA (s), KPS (t), CEA (u), and CA199 (v) in healthy volunteers and CRC patients.

*n* = 8 mice per group. One-way ANOVA followed by Tukey multiple-comparison test in mice. The p-value showed the difference that all groups were compared to PBS groups. ****: p < 0.0001, **: p < 0.01.

*n* = 40 people per group. Independent-samples test-test in people. ****: p < 0.0001, **: p < 0.01.

The fifth batch of the animal trial was designed according to Figure 8a. Three-week-old Apc^Min+^ mice were fed adaptively for 1 week. The pseudo-germ-free Apc^Min+^ mice model group (*n* = 8), was fed an ordinary diet and mixed antibiotic water (vancomycin 500 mg/L, ampicillin 1 g/L, neomycin sulfate 1 g/L, and metronidazole 1 g/L). After 4 weeks, the successfully modeled pseudo-germ-free mice were randomly divided into three groups, namely, the PBS group, FMT-CCFM683 group, and FMT-CCFM683Δ*bbi* group, with eight mice in each group. The donor mice in the corresponding group were given fresh fecal bacteria extract (100 μL/10 g) once a day. The mice in the PBS group were gavaged with 0.2 mL/day PBS.

The sixth batch of the animal trial was designed according to Figure 9a. Sixteen Apc^Min+^ mice were divided into two groups (*n* = 8 for each group) and fed with sterile water and standard chow. Briefly, the mice in the PBS group were gavaged with 0.2 mL/day PBS, and in the other group, approximately 10^9^ CFU/day of *O. splanchnicus* was gavaged to each mouse.

### 2.4. Pathological and immunological analysis

At the end of the experiment, mice were euthanized, and immediately dissected to remove the colon, colonic contents were collected, and the colon tissues were cleaned with cold PBS. The colon was then dissected longitudinally to measure the size and number of visible tumors. Portions of the colon tissue were fixed with 4% polyformaldehyde. The fixed colons were dehydrated, embedded, sectioned, and stained with hematoxylin and eosin (H&E). Histological analysis of polyps, adenomas, and adenocarcinomas was performed by a certified pathologist as previously described.^15^ The results were presented as the percentage of high-grade lesions in the colon.

Immunohistochemical staining was used to detect Ki67 and PCNA in colon tissues (rabbit anti, Abcam, Cambridge, UK). All stains were performed using horseradish peroxidase-conjugated antibodies, visualized with the 3-3′-diaminobenzidine substrate, and counterstained with hematoxylin. The mean optical density values of Ki67 and PCNA were analyzed by Image J.

### 2.5. Analysis of short-chain and long-chain fatty acids

Briefly, short-chain fatty acids (SCFAs) were extracted using a previous method and transferred to a gas chromatography vial for gas chromatography-mass spectrometry analysis.^16^

Long-chain fatty acids were extracted from mouse liver, mouse colon contents, mouse serum, and volunteer feces according to a previous method, and then methylated.^17^ The methylated solution was recovered with n-hexane, and the fatty acids were measured and analyzed by gas chromatography-mass spectrometry.^18^ The CLA production ability of CCFM683, MY40C, CCFM681, CCFM683Δ*bbi*, and CCFM683Δ*bbi:bbi in vitro* was determined as previously described.^19^

### 2.6. Analysis of the intestinal mechanical barrier

RIPA lysis buffer (Beyotime Biotechnology, Shanghai, China) was added to the colon at a ratio of 9:1 (w/w) and homogenized. Colon tissue homogenate was centrifuged at 15000 g for 15 min at 4℃, the supernatant was carefully collected and stored at -80℃.

ELISA kits (Fcmacs Biotech Co., Ltd., Nanjing, Jiangsu, China) were used to detect the concentrations of MUC2, Claudin-1, ZO-1, Occludin, Bax, and Bcl-2 in the colon tissue supernatant. The total protein concentration in the supernatant was determined using a BCA kit (Beyotime Biotechnology, Shanghai, China). Colon tissue sections were hydrated and blocked, then incubated with rabbit anti-mouse MUC2, ZO-1, Occludin, Claudin-1, Bax, and Bcl-2 antibodies (Abcam, Cambridge, UK). These proteins were then measured using a previously described method.^20^ The mean optical density values of these proteins were analyzed by Image J.

TUNEL staining of colon tissue was performed according to the instructions of the TUNEL Cell Apoptosis Detection Kit (Nanjing Vazyme Biotech Co., Ltd., Jiangsu, China). DAPI was used to stain cell nuclei. The number of TUNEL-positive cells was analyzed by Image J.

### 2.7. Cytokine analysis

Commercially available ELISA kits (Fcmacs Biotech Co., Ltd. Nanjing, Jiangsu, China) were used to detect the levels of IFN-γ, IL-6, IL-10, IL-1β, and TNF-α in the colon tissue supernatant. Multiplex immunofluorescent staining of IL-1β, TNF-α, and IL-10 was performed by Wuhan Servicebio Biotechnology Co. LTD (Wuhan, Hubei, China).

### 2.8. RNA extraction and quantitative real-time PCR

RNA in the colon tissue was extracted using a Trizol reagent (Vazyme, Jiangsu, China). The extracted RNA was reverse-transcribed into complementary DNA (cDNA) using a commercial kit (Takara, Tokyo, Japan). Quantitative PCR analysis was performed as previously described. The expression of genes was calculated using the 2^−ΔΔCt^ method.^12^ The gene primers used in this study were listed in Table S2.

### 2.9. Western blot analysis

Western blot was used to detect PPAR-γ, phosphor-p65 (p-p65), and phosphor-IκBα (p-IκBα).^21^ Colon tissue was lysed with RIPA and centrifuged at 12,000 g for 20 min to obtain the supernatant. The proteins in the supernatant were separated by 8%–12% SDS-polyacrylamide gel electrophoresis and then transferred to a PVDF membrane. The membrane was blocked with 2% bovine serum albumin for 2 h and then incubated with the corresponding primary antibodies (Abcam, Cambridge, UK) overnight at 4°C. Finally, the membrane was incubated with anti-rabbit or anti-mouse IgG antibodies, respectively (Abcam, Cambridge, UK). The grayscale values of the proteins were measured using Image J.

### 2.10. Fecal gut microbiota analysis

The total bacterial DNA was extracted from the feces, and then the V3-V4 region of the 16S rRNA gene was amplified as previously described.^22^ After sequencing, the raw data was analyzed using QIIME 2. Alpha and beta diversity and Lefse were analyzed online as previously described.^23^ Significant differences in the genus, CRC indicators, metabolites, and cytokines were analyzed for correlation using Gephi 0.9.2 and R 4.3.1.

### 2.11. Mouse fecal sample metagenomic sequencing

Shotgun metagenomic sequencing of mouse fecal samples was performed by Majorbio Bio-Pharm Technology Co., Ltd (Shanghai, China). The specific method has been mentioned in a previous study.^24^ Detailed information on gene function and taxonomy was obtained using BLASTP as previously described.^25^ Principal coordinate analysis (PCoA) was performed using Bray Curtis. The differences at the species level between the PBS group and the CCFM683 group were measured using Lefse. Data processing was performed on the Majorbio Cloud Platform (http://www.majorbio.com). Significant differences in species, CRC indicators, colonic mechanical barriers, and cytokines were analyzed for correlation using Gephi 0.9.2 and R 4.3.1.

### 2.12. Transcriptome analysis of colon tissue

The colon samples of the mice (n = 6 per group) were sent to Majorbio Biopharm Technology Co., Ltd., Shanghai, China for transcriptome sequencing and analysis. The data were analyzed on the free online platform of Majorbio Cloud Platform (www.majorbio. com).^26^ The principal component analysis (PCA) was used to verify the results of the grouping based on the gene expression levels. Normalization of read counts and differential expression analysis of genes (DEG) between sample groups were performed using DESeq2^26^. Statistical significance of the differentially expressed genes was defined with the adjusted p-value < 0.05 correcting for multiple testing. All DEGs were used to perform functional enrichment cluster based on the Kyoto Encyclopedia of Genes and Genomes (KEGG) pathway. A bubble diagram of DEGs from the PPAR signaling pathway was plotted.

### 2.13. Volunteer serum immune marker analysis

ELISA kits (Fcmacs Biotech Co., Ltd. Nanjing, Jiangsu, China) were used to measure the concentrations of PPAR-γ, TNF-α, IL-1β, and IL-10 in the serum of CRC patients and healthy volunteers. Indicators such as carcinoembryonic antigen (CEA) and cancer antigen (CA)-199 in the serum of CRC patients were analyzed through hospital physical examination. The Karnofsky Performance Scale (KPS) score sheet was independently filled out by CRC patients and healthy volunteers.

### 2.14. Statistical analysis

Data were analyzed using SPSS 23.0, GraphPad Prism 8.0, and R 4.3.1. Significant differences were analyzed by one-way ANOVA according to Tukey’s tests, independent-samples T-test, and represented by *p*-value.

## 3. Results

### 3.1. Analysis of healthy volunteers and CRC patients’ indicators

**Figure 1.**
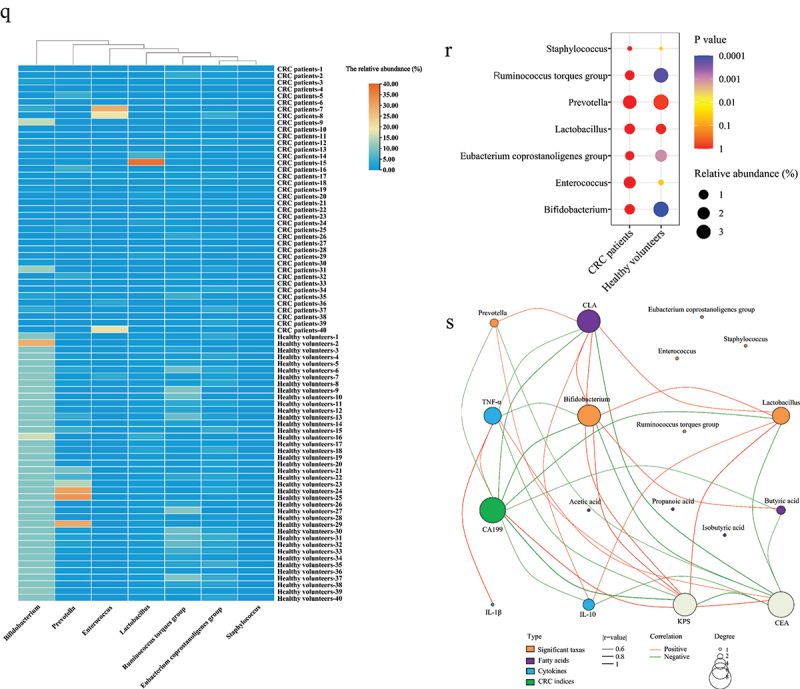
(Continued).

To screen for potential differential metabolites and gut bacteria, we analyzed the gut microbiota and metabolites of healthy volunteers and CRC patients. SCFAs and CLA in feces were analyzed. The results showed that CLA in the feces of CRC patients was 71.12% of that in healthy volunteers. Moreover, butyric acid in the feces of CRC patients was 43.26% lower than that of healthy volunteers (Figures 1a,b). However, there were no significant differences in other SCFAs between healthy volunteers and CRC patients (Figures S1A–S1D). In addition, the concentration of IL-10 in the serum of CRC patients was significantly lower than that in healthy volunteers, and the concentrations of TNF-α and IL-1β were 1.75 and 1.57 times that of healthy volunteers, respectively (Figures 1c–e).
**Figure 1.**
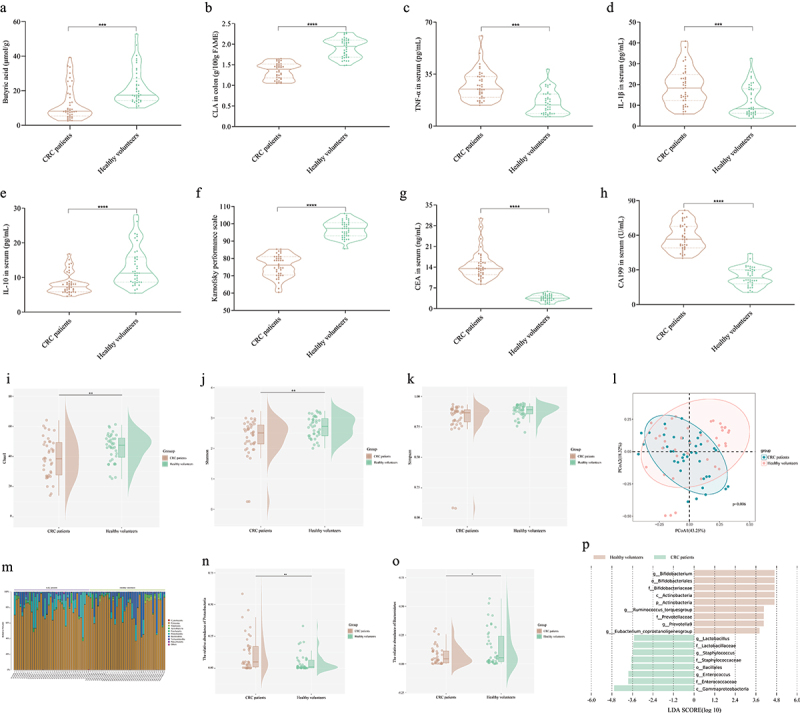
The analysis of differences between CRC patients and healthy volunteers.

(a-b) The concentration of fecal SCFAs and CLA. The concentration of TNF-α (c), IL-1β (d), and IL-10 (e). (f) The KPS score. The CEA (g) and CA199 (h) in serum. The Chao1 index (i), Shannon index (j), and Simpson index (k). (l) The β-diversity of gut microbiota. (m) The phylum level of gut microbiota. The relative abundance of Proteobacteria (n) and Bacteroidetes (o). (p) Distribution histogram based on LDA at genus level and LDA score > 3.5. (q-r) Relative abundance of differential genus. The p-value represents a discrepancy compared with the CRC patients group. (s) Correlation analysis of the significant genus, fatty acids, CRC indices, and cytokines. Only significant correlations (p < 0.05, |r^2^| > 0.6) are displayed with an edge.

*n* = 40 people per group. Independent-samples test-test. ****: p < 0.0001, ***: p < 0.001, **: p < 0.01.

In addition, we analyzed KPS scores and tumor-related indicators such as serum CEA and serum CA199. The results showed that the KPS score of healthy volunteers (96.84) was significantly higher than that of CRC patients (75.82), while the CEA and CA199 levels in the serum of healthy volunteers were 24.84% and 41.55% of CRC patients, respectively (Figures 1f–h).

In addition, the gut microbiota of healthy volunteers and CRC patients were compared. The Chao1 and Shannon indices of gut microbiota in healthy volunteers were significantly higher than those in CRC patients (Figures 1i–k). There was a significant difference in the β-diversity of the gut microbiota between healthy volunteers and CRC patients (Figure 1l). Compared with CRC patients, the relative abundance of Bacteroidetes in healthy volunteers was significantly increased, while Proteobacteria was significantly reduced (Figures 1m-o). The relative abundance of *Bifidobacterium*, *Ruminococcus torques group*, and *Eubacterium coprostanoligenes group* in the gut of healthy volunteers was significantly higher than those in CRC patients, while the relative abundance of *Enterococcus* and *Staphylococcus* was significantly lower than that in CRC patients (Figures 1p-r).

In addition, we analyzed the correlation between significantly different genera, CRC indices, cytokines, and metabolites. Butyric acid showed a significant positive correlation with CLA and a significant negative correlation with CEA and CA199 (p < 0.01). Correlation analysis showed that *Bifidobacterium* and CLA were strongly positively correlated with KPS (p < 0.001), and negatively correlated with CEA and CA199 (p < 0.001) (Figure 1s). The results suggest it may be possible to prevent CRC by supplementing with CLA-producing *Bifidobacterium*.

### 3.2. Effects of CLA-producing Bifidobacterium on CRC mice

**Figure 2.**
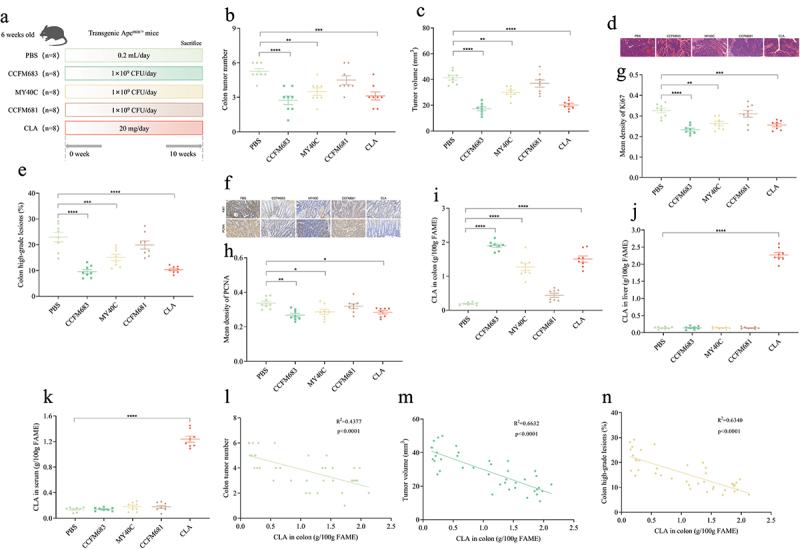
Effects of CLA-producing *Bifidobacterium* on CRC mice.

Our previous studies have shown that *B. breve* CCFM683, *B. longum* CCFM681, and *B. pseudocatenulatum* MY40C had different CLA production capabilities *in vitro*, and had different regulatory effects on intestinal immunity and mechanical barrier.^20,23,27^ Therefore, a spontaneous CRC model was constructed using C57BL/6J Apc^Min+^ mice to explore the effect of CLA-producing *Bifidobacterium* on CRC. Compared with mice treated with PBS, mice preventatively treated with CCFM683, MY40C, and CLA developed significantly smaller and significantly fewer tumors, while treatment with CCFM681 gave no significant difference (Figures 2b,c, Figure S2). The tumors in the PBS group CRC mice were pathologically identified as polyps with severe dysplasia or early cancer with submucosal infiltration. CCFM683, MY40C, and CLA prevented early cancer in the colon (Figure 2d). Compared with the PBS group, after treatments with CCFM683, MY40C, and CLA, only 9.56%, 15.07%, and 10.33%, respectively, of mice in the prevention groups were found to have high-grade dysplastic adenocarcinoma (including early tumors) in the colon, which was significantly lower than the PBS group CRC mice (22.98%), and there was no significant difference between the CCFM681 prevention group and the PBS group (Figures 2e).

(a) Experimental design for *Bifidobacterium* treatment for an Apc^Min+^ mouse model. (b) The colon tumor number. (c) The colon tumor volume. (d) HE staining for mice colons, scale bars, 50 μm. (e) The percent of high-grade dysplastic adenocarcinoma of the colon. (f) Immunohistochemical staining for Ki67 and PCNA in the colon, scale bars, 50 μm. The mean optical density values of Ki67 (g) and PCNA (h) in the colon. The CLA concentration in the feces of colon (i), liver (j), and serum (k). The interdependent quantitative relationships between the CLA concentration in colon and colon tumor number (l), colon tumor volume (m), and colon high-grade lesions (n).

*n* = 8 mice per group. One-way ANOVA followed by Tukey multiple-comparison test. The p-value showed the difference that all groups were compared to PBS groups. ****: p < 0.0001, ***: p < 0.001, **: p < 0.01.

Ki67 and PCNA are cell proliferation biomarkers, and their expression in the colon of CRC mice was detected by immunohistochemistry. Compared with the PBS group, after treatments with CCFM683, MY40C, and CLA, the expression of Ki67 and PCNA in the colon was reduced (Figures 2f), and the average optical density values of Ki67 and PCNA were significantly reduced, while there was no significant difference in the average optical density values of Ki67 and PCNA in the colon of mice in the CCFM681 prevention group compared with the PBS group (Figures 2g,h). Therefore, CCFM683 and MY40C significantly prevented CRC, while CCFM681 had no significant regulatory effect on CRC.

In addition, the effect of *Bifidobacterium* on the CLA content in CRC mice was analyzed. Compared with the PBS group, treatments with CCFM683, MY40C, CCFM681, and CLA increased the CLA content in the feces of colon by 903.81%, 575.32%, 132.42%, and 697.14%, respectively (Figures 2i). Treatments with the three *Bifidobacterium* strains did not increase the CLA content in the serum and liver, while treatment with CLA significantly increased the CLA content in the serum and liver (Figures 2j,k). The CLA concentration in the feces of colon significantly negatively correlated with the number of colon tumors, tumor volume, and high-grade lesions in the colon (Figures 2l–n), which indicated that the ability of *Bifidobacterium* to produce CLA in the feces of colon may be related to its effectiveness in relieving CRC. Therefore, CLA may be the potential metabolite for CCFM683 to prevent CRC.

### 3.3. Analysis of key metabolite regulated by CCFM683 in CRC

**Figure 3.**
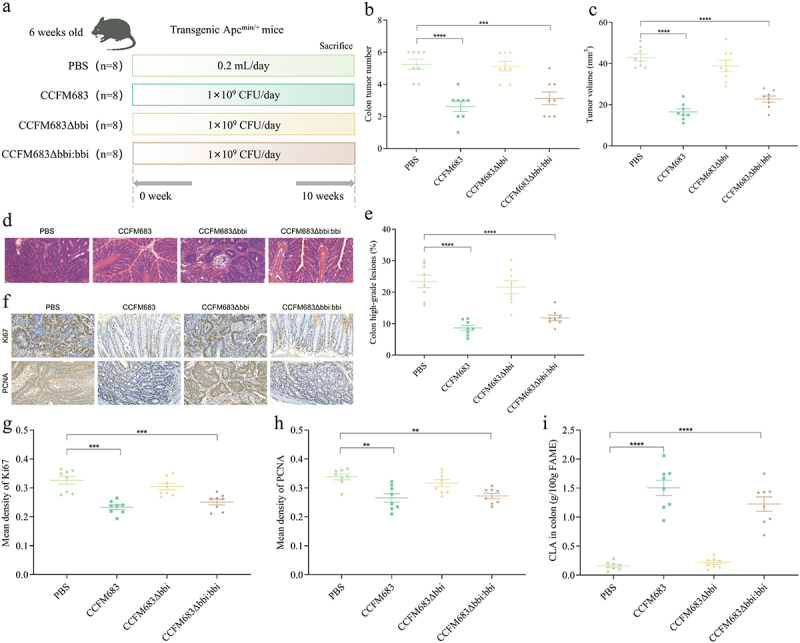
Analysis of key metabolite of CCFM683 in relieving CRC.

Previous research has found that CCFM683 can convert LA to CLA via bifidobacterial linoleic acid isomerase (*bbi*).^11^ To clarify the role of CLA in mitigating CRC in CCFM683, we constructed a linoleic acid isomerase knockout mutant CCFM683Δ*bbi* and its corresponding complementation CCFM683Δ*bbi:bbi*. Based on *in vitro* tests, the CLA conversion ratio of CCFM683 and CCFM683ΔΔ*bbi:bbi* was 92.25% and 85.62%, respectively, while that of CCFM683Δ*bbi* was 0% (Table S3). Then, we used CCFM683Δ*bbi* and CCFM683Δ*bbi:bbi* to intervene in CRC mice to determine the role of CLA in CRC mitigation. Compared with the PBS group, the number and volume of tumors in the colon of CRC mice were significantly reduced after treatments with CCFM683 and CCFM683Δ*bbi:bbi*, while there was no significant change after treatment with CCFM683Δ*bbi* (Figures 3b,c, Figure S3A). Orally administered CCFM683 and CCFM683Δ*bbi:bbi* prevented the severe dysplastic polyps in the colon but administration of CCFM683Δ*bbi* was similar to the PBS group (Figure 3d). Compared with the PBS group, after treatments with CCFM683 and CCFM683Δ*bbi:bbi*, 8.64% and 11.84% of the colons, respectively, were found to have high-grade dysplastic adenocarcinoma (including early tumors), which was significantly lower than the PBS group (23.43%), and there was no significant difference between the CCFM683Δ*bbi* prevention group and the PBS group (Figure 3e).

(a) Experimental design for CCFM683, CCFM683Δ*bbi*, and CCFM683Δ*bbi:bbi* treatments for an Apc^Min+^ mouse model. (b) The colon tumor number. (c) The colon tumor volume. (d) HE staining for mice colons, scale bars, 50 μm. (e) The percent of high-grade dysplastic adenocarcinoma of the colon. (f) Immunohistochemical staining for Ki67 and PCNA in the colon, scale bars, 50 μm. The mean optical density values of Ki67 (g) and PCNA (h) in the colon. The CLA concentration in the feces of colon (i).

*n* = 8 mice per group. One-way ANOVA followed by Tukey multiple-comparison test. The p-value showed the difference that all groups were compared to PBS groups. ****: p < 0.0001, ***: p < 0.001, **: p < 0.01.

In addition, compared with the PBS, after treatments with CCFM683 and CCFM683Δ*bbi:bbi*, the expression levels of Ki67 and PCNA in the colon were reduced (Figure 3f), and the average optical density values of Ki67 and PCNA were significantly reduced, while there was no significant difference in the average optical density values of Ki67 and PCNA in the colon of mice in the CCFM683Δ*bbi* prevention group compared with the PBS group (Figures 3g,h). Therefore, CLA was a crucial metabolite for CCFM683 to prevent CRC.

In addition, the concentrations of CLA in the feces of colon of mice treatments with CCFM683 and CCFM683Δ*bbi:bbi* were 9.48 and 7.71 times that of mice treatment with PBS, respectively, while there was no significant difference in the CLA content in the feces of colon of mice in the CCFM683Δ*bbi* prevention group compared with the PBS group (Figure 3i). In addition, compared with the PBS group, treatments with CCFM683, CCFM683Δ*bbi*, and CCFM683Δ*bbi:bbi* did not increase the CLA content in the serum and liver (Figures S3B and S3C).

### 3.4. Effects of bbi on preventing CRC

**Figure 4.**
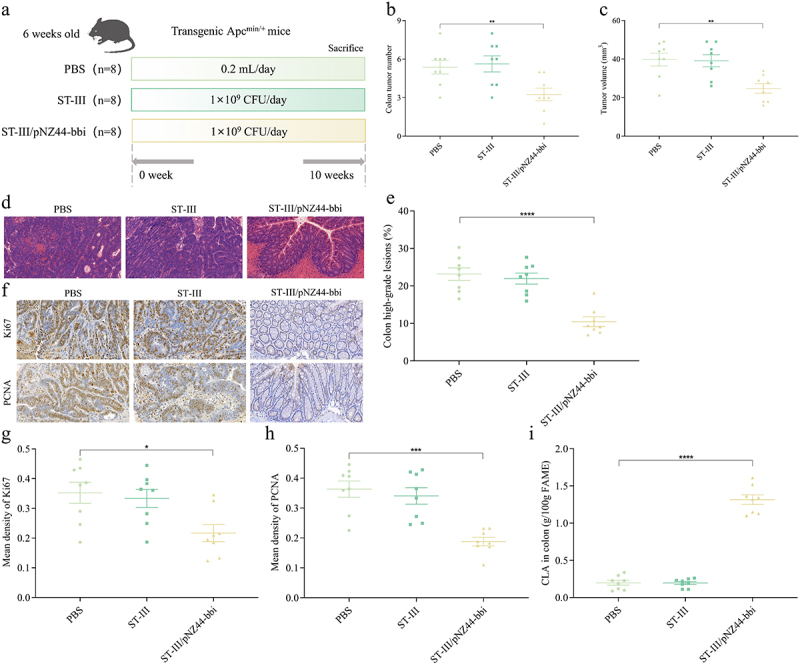
Effects of *bb*i gene on CRC.

To clarify the role of *bbi* in preventing CRC, we used the previous method to introduce the *bbi* gene into *L. plantarum* ST-III, which does not contain the *bbi* gene and does not produce CLA, and obtained the engineered strain ST-III/pNZ44-*bbi*. The CLA conversion rates of ST-III and ST-III/pNZ44-*bbi* were 0 and 78.64% *in vitro*, respectively (Table S3). We used ST-III and ST-III/pNZ44-*bbi* to intervene in CRC mice to determine the role of *bbi* in CRC mitigation. Compared with the PBS group, the number and volume of tumors in the colon of CRC mice were significantly reduced after treatment with ST-III/pNZ44-*bbi*, while there was no significant change after treatment with ST-III (Figures 4b,c, Figure S4A). Orally administered with ST-III/pNZ44-*bbi* prevented the severe dysplastic polyps in the colon, while the treatment with ST-III was similar to the PBS group (Figure 4d). Compared with the PBS group, after treatment with ST-III/pNZ44-*bbi*, 10.45% of the colons were found to have high-grade dysplastic adenocarcinoma (including early tumors), which was significantly lower than the PBS group (23.15%), and there was no significant difference between the ST-III prevention group and the PBS group (Figure 4e).

(a) Experimental design for ST-III and ST-III/pNZ44-*bbi* treatments for an Apc^Min+^ mouse model. (b) The colon tumor number. (c) The colon tumor volume. (d) HE staining for mice colons, scale bars, 50 μm. (e) The percent of high-grade dysplastic adenocarcinoma of the colon. (f) Immunohistochemical staining for Ki67 and PCNA in the colon, scale bars, 50 μm. The mean optical density values of Ki67 (g) and PCNA (h) in the colon. The CLA concentration in the feces of colon (i).

*n* = 8 mice per group. One-way ANOVA followed by Tukey multiple-comparison test. The p-value showed the difference that all groups were compared to PBS groups. ****: p < 0.0001, ***: p < 0.001, **: p < 0.01.

In addition, compared with the PBS treatment, the expression levels of Ki67 and PCNA in the colon were reduced after treatment with ST-III/pNZ44-*bbi* (Figure 4f), and the average optical density values of Ki67 and PCNA were significantly reduced, while there was no significant difference in the average optical density values of Ki67 and PCNA in the colon of mice in the ST-III prevention group compared with the PBS group (Figures 4g,h). Therefore, the *bbi* gene plays an important role in preventing CRC.

In addition, CLA in the feces of colon of mice treatment with ST-III/pNZ44-*bbi* was 6.65 times that of mice treatment with PBS, while there was no significant difference in the CLA content in the feces of colon of mice in the ST-III prevention group compared with the PBS group (Figure 4i). Also, compared with the PBS group, treatments with ST-III and ST-III/pNZ44-*bbi* did not increase the CLA content in the serum and liver (Figures S4B and S4C).

### 3.5. Analysis of key receptors regulated by CCFM683 in CRC

RNA-sequencing on colon tissue was performed to test the differential expression of genes between PBS and CCFM683 treatments. There were differences in the transcriptional profiles of mice in the different groups with principal component analysis (PCA) (Figure 5a), indicating that CCFM683 treatment altered the transcription profile of CRC mice. PBS and CCFM683 groups share 14,348 genes, while PBS and CCFM683 were unique to 378 and 569 genes, respectively (Figure 5b). Compared with the CCFM683 treatment, PBS significantly up-regulated 66 genes and down-regulated 288 genes (Figure 5c). When mapped to the KEGG pathway database, these genes showed enrichment in a series of metabolic signaling pathways, especially those related to fatty acid metabolism and lipid metabolism (Figure 5d). Of note, PPAR signaling was the most significantly enriched signaling pathway in CCFM683-treated mice (Figure 5d). Six genes that were enriched in the PPAR signaling pathway were mainly involved in lipid metabolism (PPARg), and the endocrine system (Apoa1, Fabp6, Apoc3, Acox2, and Aqp7) (Figures 5e,f), especially the most significantly up-regulated PPARg in CCFM683-treated mice (Figures 5e-f). Overall, these results suggested that CCFM683 affected the expression of genes related to lipid metabolism and endocrine system and the activation of PPAR signaling may contribute to reflect CRC prevention. Compared with the PBS group, treatments with CCFM683 and CCFM683Δ*bbi:bbi* significantly increased the mRNA expression and protein concentration of PPAR-γ, while treatment with CCFM683Δ*bbi* had no significant effect on PPAR-γ (Figures 5d-i). Thus, PPAR-γ may be an important receptor for CCFM683 to prevent CRC.
**Figure 5.**
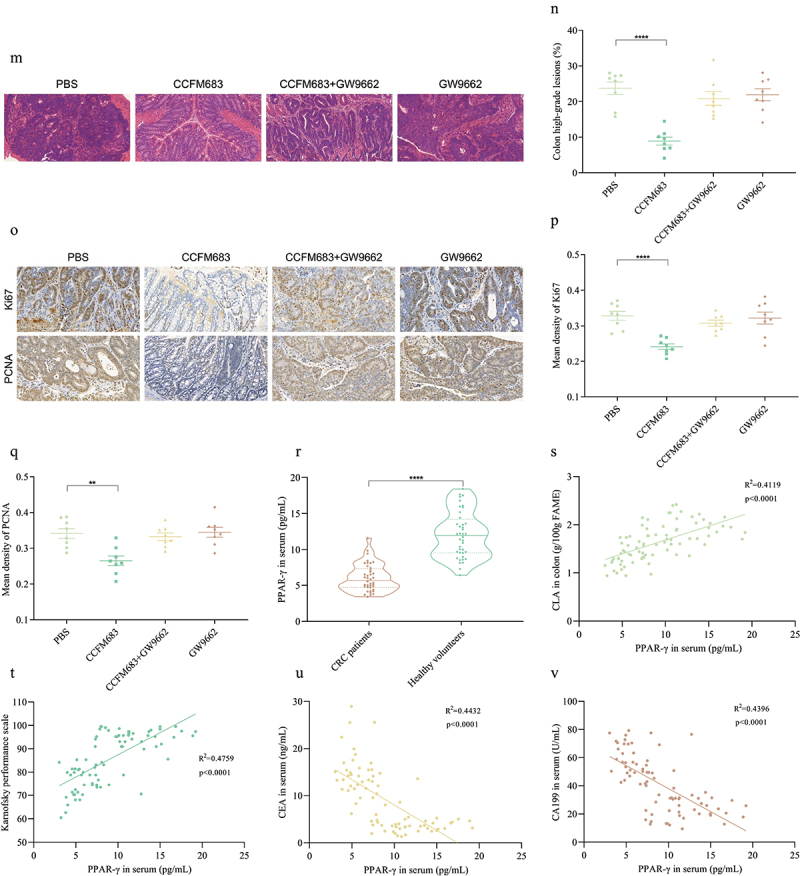
(Continued).

Based on this, we treated CCFM683-intervened CRC mice with a PPAR-γ inhibitor (GW9662) to clarify the role of PPAR-γ. Compared with the PBS group, after treatment with CCFM683, the number and volume of tumors in the colon of CRC mice were significantly reduced, while there was no significant change after treatments with CCFM683+GW9662 and GW9662 (Figures 5k,l, Figure S5). Orally administered CCFM683 prevented the severe dysplastic polyps in the colon, while orally administered CCFM683+GW9662 or GW9662 were similar to the PBS group (Figure 5m). Compared with the PBS group, 8.90% of colons in the CCFM683 group were found to have high-grade dysplastic adenocarcinoma (including early tumors), which was significantly lower than the PBS group (23.75%), while there was no significant difference among CCFM683+GW9662, GW9662 and PBS groups (Figure 5n).

In addition, compared with the PBS group, the expression levels of Ki67 and PCNA in the colon after treatment with CCFM683 were reduced (Figure 5o), and the average optical density values of Ki67 and PCNA were significantly reduced, while there was no significant difference in the average optical density values of Ki67 and PCNA in the colon of mice in the CCFM683+GW9662 or GW9662 groups compared with the PBS group (Figures 5p,q). Further, the concentration of PPAR-γ in the serum of healthy volunteers and CRC patients was compared. The concentration of PPAR-γ in the serum of healthy volunteers was 1.95 times that of CRC patients (Figure 5r). PPAR-γ significantly negatively correlated with CEA and CA19-9, while significantly positively correlated with KPS and CLA (Figures 5s-v). Therefore, PPAR-γ is an important receptor for CCFM683 to prevent CRC.

### 3.6. Effects of CCFM683 on the intestinal mechanical barrier in CRC mice

**Figure 6.**
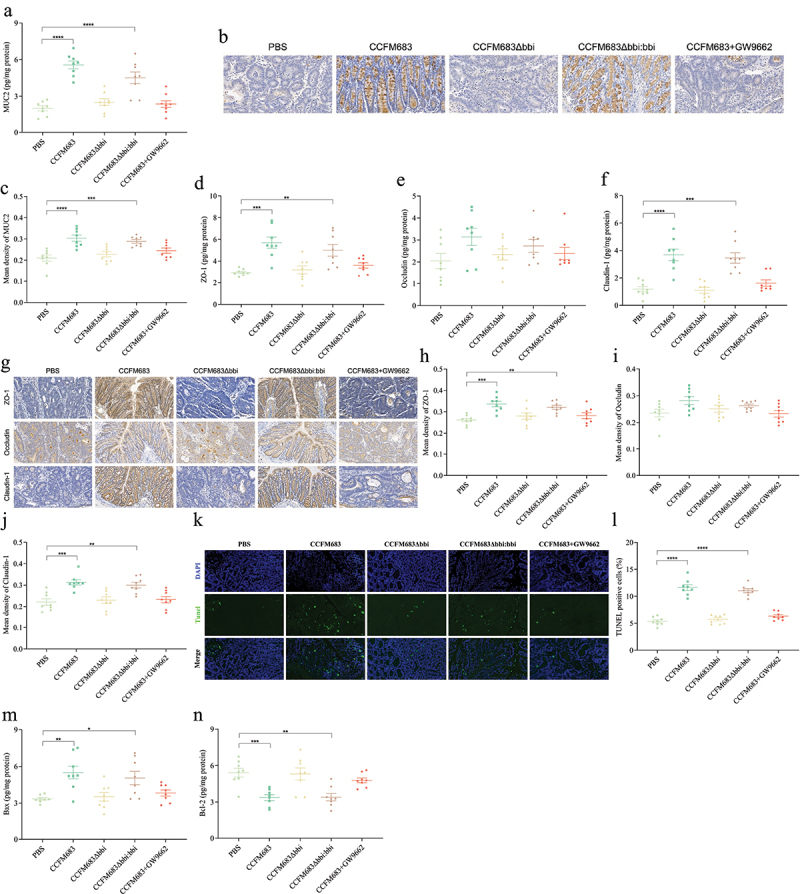
Effects of CCFM683 on the intestinal mechanical barrier in CRC mice.

To assess the impact of CCFM683 on the colonic mucous layer, the concentration of MUC2 was determined. Treatments with CCFM683 and CCFM683Δ*bbi:bbi* significantly increased the concentration of MUC2, while its concentration in CCFM683Δ*bbi* and CCFM683+GW9662 treated mice was similar to that in PBS-treated mice (Figure 6a). Immunohistochemical analysis of MUC2 yielded similar results (Figures 6b,c). Therefore, improving the intestinal mucous layer was one way to prevent CRC for CCFM683.

(a) The concentration of MUC2 in the colon. (b) Immunohistochemical staining for MUC2 in the colon, scale bars, 50 μm. (c) The mean optical density values of MUC2. The concentration of ZO-1 (d), Occludin (e), and Claudin-1 (f) in the colon. (g) Immunohistochemical staining for ZO-1, Occludin, and Claudin-1, scale bars, 50 μm. The mean optical density values of ZO-1 (h), Occludin (i), and Claudin-1 (j). (k) TUNEL staining for colon, scale bars, 50 μm. (l) The percent of TUNEL-positive cells. The concentration of Bax (m) and Bcl-2 (n) in the colon.

*n* = 8 mice per group. One-way ANOVA followed by Tukey multiple-comparison test. The p-value showed the difference that all groups were compared to PBS groups. ****: p < 0.0001, ***: p < 0.001, **: p < 0.01, *: p < 0.05.

To evaluate the impact of CCFM683 on colonic tight junctions, tight junction (TJ) proteins were measured. Compared with PBS group mice, treatments with CCFM683 and CCFM683Δ*bbi:bbi* significantly increased the concentration of ZO-1 and Claudin-1 in the colon, while treatments with CCFM683Δ*bbi* or CCFM683+GW9662 had no significant effect on ZO-1 and Claudin-1 (Figures 6d,f). Additionally, there was no significant difference in Occludin among all groups (Figure 6e). Interestingly, immunohistochemical analysis of these TJ proteins yielded similar results (Figures 6g-j). Therefore, up-regulating intestinal TJ proteins is an important pathway for CCFM683 to prevent CRC.

Additionally, the impact of CCFM683 on colonic epithelial cell apoptosis was determined. Compared with the colon of PBS group mice, treatments with CCFM683 and CCFM683Δ*bbi:bbi* significantly promoted apoptosis of colonic epithelial cells, while treatments with CCFM683Δ*bbi* or CCFM683+GW9662 had no significant effect on epithelial cell apoptosis (Figures 6k-l). Furthermore, treatments with CCFM683 and CCFM683Δ*bbi:bbi* significantly increased the concentration of the pro-apoptotic protein Bax and reduced the anti-apoptotic protein Bcl-2, while treatments with CCFM683Δ*bbi* or CCFM683+GW9662 had no significant effect on these proteins (Figures 6m,n) and the immunohistochemical analysis showed similar results (Figures S6A-6C). Therefore, promoting apoptosis of intestinal epithelial cells in CRC mice is an important pathway for CCFM683 to prevent CRC.

### 3.7. Effects of CCFM683 on cytokines and signaling pathways in CRC mice

**Figure 7.**
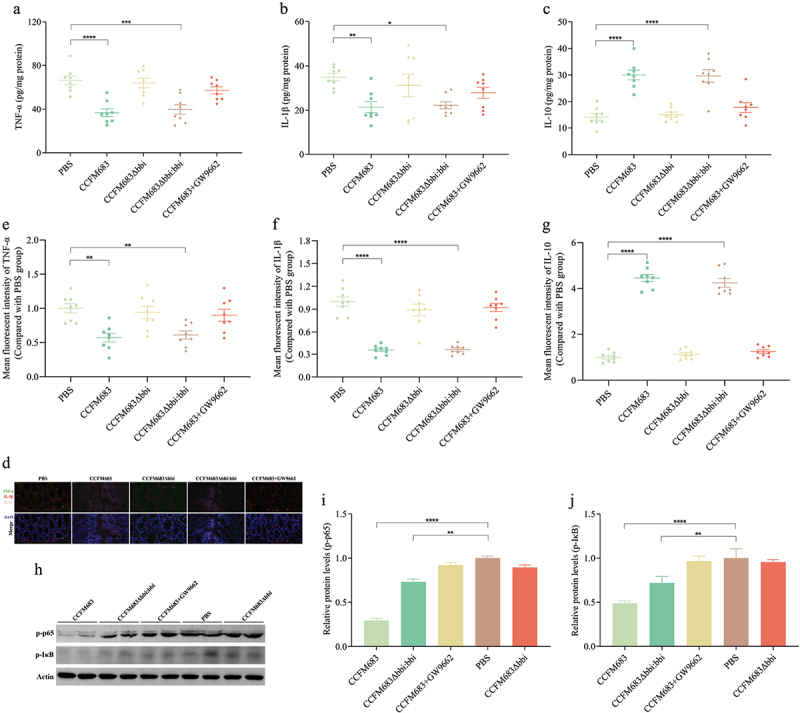
Effects of CCFM683 on the intestinal immune barrier in CRC mice.

Compared with the PBS group, treatments with CCFM683 and CCFM683Δ*bbi:bbi* significantly reduced TNF-α and IL-1β in the colon and significantly increased IL-10, while there was no significant effect on IL-6 and IFN-γ (Figures 7a-c, Figures S7A and S7B). However, there was no significant difference in these five cytokines in the colon of mice in the CCFM683Δ*bbi* and CCFM683+GW9662 groups compared with the PBS group. Multiple immunofluorescence staining showed that compared with PBS group CRC mice, treatments with CCFM683 and CCFM683Δ*bbi:bbi* significantly reduced TNF-α and IL-1β and increased IL-10 (Figures 7d-g).

The concentration of TNF-α (a), IL-1β (b), and IL-10 (c) in colonic tissue. (d) Multiple immunofluorescence staining for TNF-α, IL-1β, and IL-10 in the colon, scale bars, 20 μm. The mean fluorescent intensity of TNF-α (e), IL-1β (f), and IL-10 (g). (h) Western blot of p-p65 and p-IκB. Relative p-p65 (i) and p-IκB (j) proteins expression in the colon.

*n* = 8 mice per group. One-way ANOVA followed by Tukey multiple-comparison test. The p-value showed the difference that all groups were compared to PBS groups. ****: p < 0.0001, ***: p < 0.001, **: p < 0.01, *: p < 0.05.

To analyze the impact of CCFM683 on the intestinal NF-κB signaling pathway, the concentrations of key proteins p-p65 and p-IκB were analyzed. Compared with PBS prevention, orally administered CCFM683 and CCFM683Δ*bbi:bbi* significantly reduced the protein expression of p-p65 and p-IκB, while orally administered CCFM683Δ*bbi* or CCFM683+GW9662 had no significant effect on p-p65 and p-IκB (Figures 7h-j). Therefore, CCFM683 prevention significantly inhibiting the NF-κB signaling pathway.

### 3.8. Effects of FMT on CRC mice

**Figure 8.**
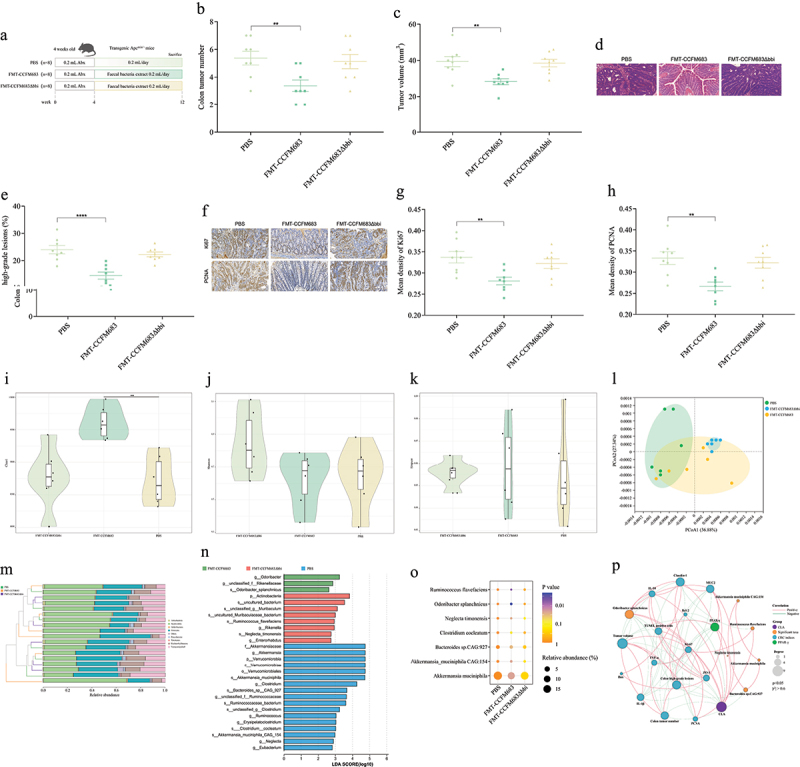
Effects of FMT on CRC mice.

Previous studies have shown that CLA and CCFM683 affected gut microbiota. Thus, the role of gut microbiota in CCFM683 prevention of CRC was investigated using pseudo-germ-free mice and FMT techniques. Compared with the PBS group, the number and volume of tumors in the colon of CRC mice were significantly reduced after treatment with FMT-CCFM683, while there was no significant change after treatment with FMT-CCFM683Δ*bbi* (Figures 8b,c, Figure S8A). Orally administered FMT-CCFM683 prevented the severe dysplastic polyps in the colon, while administration of FMT-CCFM683Δ*bbi* showed similar results to that in PBS group (Figure 8d). Compared with the PBS group, 14.46% of the colons, were found to have high-grade dysplastic adenocarcinoma (including early tumors) after treatment with FMT-CCFM683, which was significantly lower than the PBS group (23.98%), and there was no significant difference between the FMT-CCFM683Δ*bbi* prevention group and the PBS group (Figure 8e).

(a) Experimental design for FMT-CCFM683 and FMT-CCFM683Δ*bbi* treatments for an Apc^Min+^ mouse model. (b) The colon tumor number. (c) The colon tumor volume. (d) HE staining for mice colons, scale bars, 50 μm. (e) The percent of high-grade dysplastic adenocarcinoma of the colon. (f) Immunohistochemical staining for Ki67 and PCNA in the colon, scale bars, 50 μm. The mean optical density values of Ki67 (g) and PCNA (h) in the colon. The Chao1 index (i), Shannon index (j), and Simpson index (k). (l) The β-diversity of gut microbiota. (m) The phylum level of gut microbiota. (n) Distribution histogram based on LDA in species level, LDA score > 2.5. (o) Relative abundance of differential species. The p-value represents a discrepancy compared with the PBS group. (p) Correlation analysis of the significant species, CRC indices, epithelial barrier, cytokines, and apoptosis-related proteins. Only significant correlations (p < 0.05, |r^2^| > 0.6) are displayed with an edge.

*n* = 6-8 mice per group. One-way ANOVA followed by Tukey multiple-comparison test. The p-value showed the difference that all groups were compared to PBS groups. ****: p < 0.0001, ***: p < 0.001, **: p < 0.01, *: p < 0.05.

In addition, compared with the PBS group, after treatment with FMT-CCFM683, the expression levels of Ki67 and PCNA in the colon were reduced (Figure 8f), and the average optical density values of Ki67 and PCNA were significantly reduced, while there was no significant difference in the average optical density values of Ki67 and PCNA in the colon of mice in the FMT-CCFM683Δ*bbi* prevention group compared with the PBS group (Figures 8g,h). Therefore, gut microbiota played an important role in the prevention of CRC by CCFM683.

The Chao1 index of FMT-CCFM683-treated mice was significantly higher than that of PBS-treated mice, and there was no significant difference between the FMT-CCFM683Δ*bbi* prevention group and the PBS group (Figures 8i-k). Moreover, there was a significant difference in the β-diversity of gut microbiota between the FMT-CCFM683 group and the PBS group, while FMT-CCFM683Δ*bbi* group showed significant difference compared with PBS group in the β-diversity of gut microbiota (Figure 8l). Compared with the PBS and FMT-CCFM683Δ*bbi* groups, FMT-CCFM683 treatment increased the relative abundance of Bacteroidetes and decreased the relative abundance of Firmicutes (Figure 8m, Figures S8B and S8C). Differences at the species level were analyzed using Lefse. Compared with the PBS group, the abundance of *O. splanchnicus* was increased in the FMT-CCFM683 group (p < 0.001), whereas the relative abundances of *Akkermansia muciniphila*, *Bacteroides* sp.CAG:927, *A. muciniphila* CAG:154, *Neglecta timonensis*, and *Ruminococcus flavefaciens* were significantly decreased (p < 0.05), but there was no significant difference between the FMT-CCFM683Δ*bbi* prevention group and the PBS group (Figures 8n,o).

In addition, *O. splanchnicus* showed a significant positive correlation with MUC2, ZO-1, Claudin, and IL-10, and a significant negative correlation with colonic tumor numbers, tumor volumes, advanced colonic lesions, TNF-α, and IL-1β (Figure 8p). *Bacteroides* sp. CAG:927 was significantly positively correlated with colonic tumor numbers and advanced colonic lesions. However, *R. flavefaciens* was significantly negatively correlated with Claudin-1 and positively correlated with PCNA and Ki67 (Figure 8p). Based on this, we speculated that *O. splanchnicus* was a potential key bacterium for preventing CRC.

### 3.9. Effects of O. splanchnicus on CRC mice

**Figure 9.**
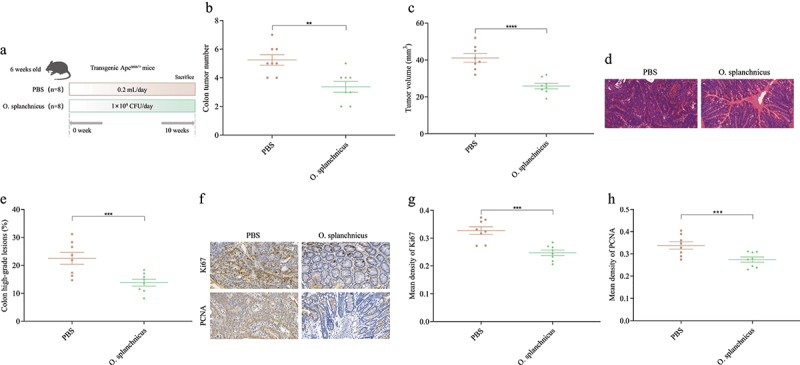
Effects of *O. splanchnicus* on CRC mice.

A spontaneous CRC model was constructed using C57BL/6J Apc^Min+^ mice, and then the impact of *O. splanchnicus* on CRC was explored. Compared with the PBS group, the number and volume of tumors in the colon of CRC mice decreased significantly after treatment with *O. splanchnicus* (Figures 9b,c, Figure S9). Tumors in the colon of PBS group CRC mice were pathologically identified as severe dysplastic polyps or early carcinomas with submucosal infiltration. Orally administered *O. splanchnicus* treated early carcinomas in the colon (Figure 9d). Only 13.81% of mice in the *O. splanchnicus* group had high-grade dysplastic adenocarcinomas (including early tumors) in the colon, significantly lower than the PBS group (22.48%) (Figure 9e). In addition, compared with PBS group CRC mice, the expression levels of Ki67 and PCNA in the colon were decreased after treatment with *O. splanchnicus* (Figure 9f), with the average optical density values of Ki67 and PCNA decreasing by 24.40% and 18.74%, respectively (Figures 9g,h). Therefore, *O. splanchnicus* can significantly prevent CRC.

(a) Experimental design for *O. splanchnicus* treatment for an Apc^Min+^ mouse model. (b) The colon tumor number. (c) The colon tumor volume. (d) HE staining for mice colons, scale bars, 50 μm. (e) The percent of high-grade dysplastic adenocarcinoma of the colon. (f) Immunohistochemical staining for Ki67 and PCNA in the colon, scale bars, 50 μm. The mean optical density values of Ki67 (g) and PCNA (h) in the colon.

*n* = 8 mice per group. Independent-samples test-test. ****: p < 0.0001, ***: p < 0.001, **: p < 0.01.

**Figure 10.**
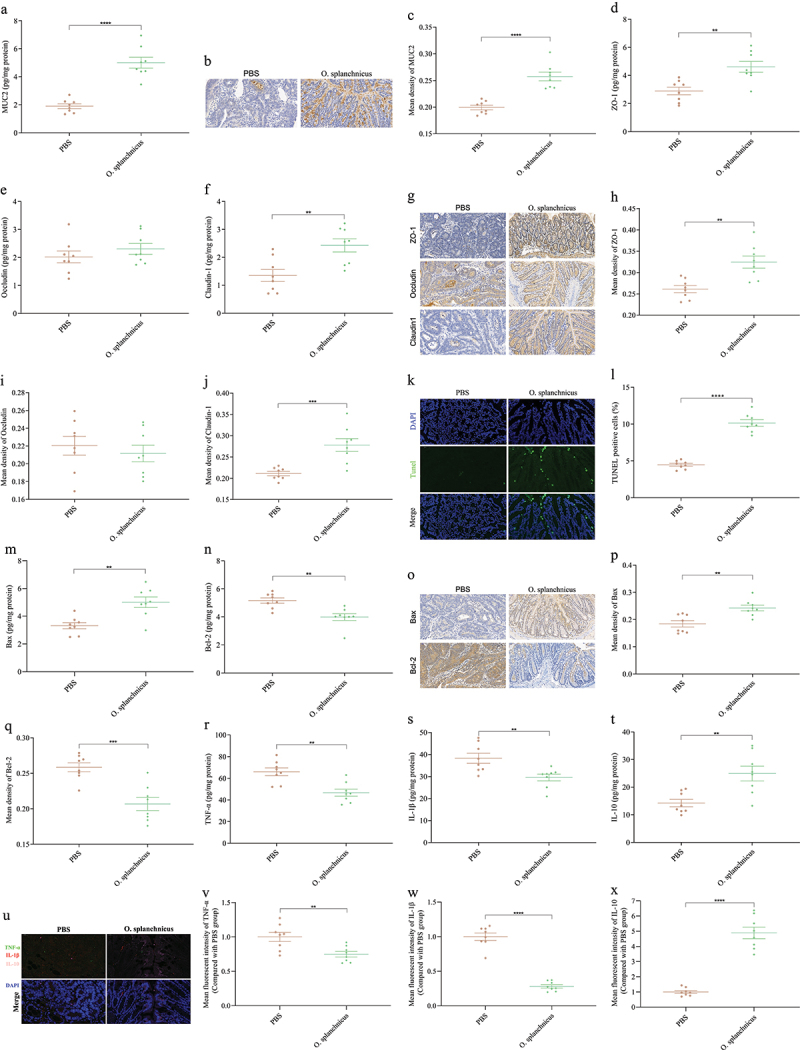
Effects of *O. splanchnicus* on intestinal mechanical barrier and immune barrier in CRC mice.

Treatment with *O. splanchnicus* significantly increased the concentration and average optical density values of MUC2 (Figures 10a-c). Compared with PBS group mice, *O. splanchnicus* treatment significantly increased the concentration and average optical density of ZO-1 and Claudin-1 in the colon (Figures 10d–j). Additionally, there was no significant difference in Occludin among all groups (Figures 10e,i). Additionally, the impact of *O. splanchnicus* on colonic epithelial cell apoptosis was determined. Compared with the colon of PBS group mice, treatment with *O. splanchnicus* significantly increased the concentration and average optical density values of the pro-apoptotic protein Bax, while the concentration and average optical density values of the anti-apoptotic protein Bcl-2 were reduced (Figures 10k–q). Therefore, *O. splanchnicus* prevented CRC by improving the mucous layer, up-regulating intestinal TJ proteins, and promoting epithelial cell apoptosis.

(a) The concentration of MUC2 in the colon. (b) Immunohistochemical staining for MUC2 in the colon, scale bars, 50 μm. (c) The mean optical density values of MUC2. The concentration of ZO-1 (d), Occludin (e), and Claudin-1 (f) in the colon. (g) Immunohistochemical staining for ZO-1, Occludin, and Claudin-1, scale bars, 50 μm. The mean optical density values of ZO-1 (h), Occludin (i), and Claudin-1 (j). (k) TUNEL staining for colon, scale bars, 50 μm. (l) The percent of TUNEL-positive cells. The concentration of Bax (m) and Bcl-2 (n) in the colon. (o) Immunohistochemical staining for Bax and Bcl-2. The mean optical density values of Bax (p) and Bcl-2 (q). The concentration of TNF-α (r), IL-1β (s), and IL-10 (t) in colonic tissue. (u) Multiple immunofluorescence staining for TNF-α, IL-1β, and IL-10 in the colon, scale bars, 20 μm. The mean fluorescent intensity of TNF-α (v), IL-1β (w), and IL-10 (x).

*n* = 8 mice per group. Independent-samples test-test. ****: p < 0.0001, ***: p < 0.001, **: p < 0.01.

Compared with the PBS group, treatment with *O. splanchnicus* significantly reduced TNF-α and IL-1β in the colon and significantly increased IL-10, while there was no significant effect on IL-6 and IFN-γ (Figures 10r-x, Figures S10A and 10B). Furthermore, the content of SCFAs in the colon of CRC mice was analyzed. Compared with the PBS group, treatment with *O. splanchnicus* significantly increased butyric acid in the colon, while the treatment with *O. splanchnicus* did not increase the content of other SCFAs such as acetic acid, propionic acid, pentanoic acid, and isopentanoic acid in the colon (Figures S11A-S11E). Therefore, gut microbiota-derived butyric acid may be the potential metabolite to prevent CRC.

**Figure 11.**
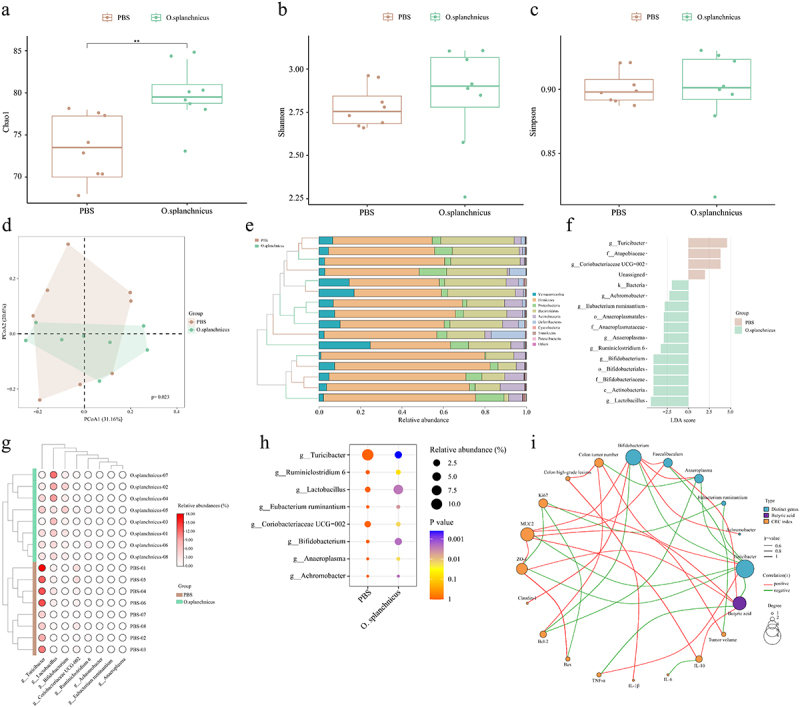
Effects of *O. splanchnicus* on gut microbiota in CRC mice.

The Chao1 index of *O. splanchnicus*-treated mice was significantly higher than that of PBS-treated mice (Figures 11a-c). There was a significant difference in the β-diversity of gut microbiota between the *O. splanchnicus* group and the PBS group (Figure 11d). Compared with the PBS group, *O. splanchnicus* treatment increased the relative abundance of Verrucomicrobia and Firmicutes, and decreased the relative abundance of Proteobacteria and Bacteroidetes (Figure 11e, Figures S11F-S11I). Differences at the genus level were analyzed using Lefse. Compared with the PBS group, the relative abundance of *Achromobacter*, *Eubacterium ruminantium*, *Anaeroplasm*, *Bifidobacterium*, and *Lactobacillus* were significantly increased in the *O. splanchnicus* group, whereas the relative abundances of *Turicibacter* and *Coriobacteriaceae* UCG-002 were significantly decreased (Figures 11f-h).

The Chao1 index (a), Shannon index (b), and Simpson index (c). (d) The β-diversity of gut microbiota. (e) The phylum level of gut microbiota. (f) Distribution histogram based on LDA in species level, LDA score > 2.0. (g-h) Relative abundance of differential genus. The p-value represents a discrepancy compared with the PBS group. (i) Correlation analysis of the significant genus, CRC indices, and butyric acid. Only significant correlations (p < 0.05, |r^2^| > 0.6) are displayed with an edge.

*n* = 8 mice per group. Independent-samples test-test. ****: p < 0.0001, ***: p < 0.001, **: p < 0.01.

In addition, the correlations among significantly different genera, CRC indicators, and SCFAs were analyzed. *Bifidobacterium* showed a significant positive correlation with MUC2, ZO-1, Claudin-1, and a significant negative correlation with Bcl-2 (Figure 11i). *Turicibacter* was significantly positively correlated with colonic tumor numbers and TNF-α, but significantly negatively correlated with MUC2, ZO-1, and butyric acid. However, *Faecalibaculum* was significantly negatively correlated with tumor volume and positively correlated with MUC2 and butyric acid. Butyric acid showed a significant positive correlation with ZO-1, MUC2, and IL-10, and a significant negative correlation with TNF-α and *Turicibacter* (Figure 11i).

## 4. Discussion

This study found that the fecal content of CLA and butyric acid in CRC patients was significantly reduced compared with healthy volunteers. It has been reported that a high intake of CLA can reduce the risk of CRC in the human body.^28^ CLA prevention can also reduce the number of intestinal polyps in Apc^min+^ mice,^29^ indicating that CLA is a potential substance for preventing CRC. Both cellular and animal experimental studies have shown that butyric acid has a regulatory effect on CRC.^30^ In addition, the relative abundance of *Bifidobacterium* was significantly lower in CRC patients compared with healthy volunteers. Correlation analysis found that CLA, butyric acid, and *Bifidobacterium* significantly negatively correlated with the severity of CRC, indicating that these indicators have potential regulatory effects on CRC. Notably, long-term consumption of 1% CLA can cause hyperinsulinemia and fat atrophy, affecting liver glutamine transaminase, liver fatty acid synthesis, and oxidation.^31,32^ Therefore, researchers are trying to produce CLA in the colon using probiotics to avoid these potential side effects. Based on this, we considered intervening CRC by supplementing CLA-producing *Bifidobacterium*, which not only supplements probiotics but also avoids the side effects of long-term CLA use.

Hence, we screened the strains *B. breve* CCFM683 and *B. pseudocatenulatum* MY40C, which significantly prevented CRC in mice. However, the key metabolites, key receptors, key pathways, and key gut microorganisms for preventing CRC were not clear. Compared with the PBS group, CCFM683, MY40C, and CLA treatments significantly increased the content of CLA in the feces of colon. CLA prevention can inhibit the proliferation of CRC cells, and promote the apoptosis of CRC cells,^33^ and CLA also showed an anti-tumor effect in CRC mice.^34^ In addition, our results showed that the concentration of CLA in the feces of colon was significantly negatively correlated with CRC. Thus, we speculated that CLA may be an important substance for CCFM683 and MY40C to prevent CRC.

CCFM683 was selected to investigate the key metabolites and potential mechanisms for preventing CRC. The results indicated that CLA was the key metabolite for CCFM683 to prevent CRC. Dietary supplementation with 1% CLA can reduce the number of colonic polyps in Apc^min/+^ mice,^29^ and in this study, orally administered CLA also significantly prevented CRC. In addition, the CLA-producing probiotic VSL#3 showed a preventing effect on CRC,^35^ which was consistent with the current study. To clarify the role of *bbi* in preventing CRC, we further introduced the *bbi* gene into *L. plantarum* ST-III which did not produce CLA to obtain the engineered strain ST-III/pNZ44-*bbi*. ST-III had no significant effect on CRC mice, while ST-III/pNZ44-*bbi* significantly prevented CRC. Therefore, the *bbi* gene can regulate the production of CLA and prevent CRC, which suggested that CRC can be prevented by constructing engineered strains containing *bbi* genes in the future.

CRC involves changes in the expression of genes related to various signaling pathways.^36^ A comprehensive analysis of transcriptome differences showed that CCFM683 had effects on PPAR-γ associated with CRC. PPAR-γ is an important anti-inflammatory mediator in the intestinal inflammatory response process.^37^ Treatments with CCFM683 and CCFM683Δ*bbi:bbi* significantly up-regulated PPAR-γ, while CCFM683Δ*bbi* had no significant effect on PPAR-γ. Therefore, CCFM683 can activate PPAR-γ by producing CLA. PPAR-γ has been reported to have anti-tumor effects, and a decrease in PPAR-γ expression promotes the formation of colon tumors.^38^ Based on these results, we speculated that PPAR-γ may be a potential receptor for CCFM683 to exert its anti-CRC effects. Inhibitor intervention results showed that PPAR-γ was the key receptor for CCFM683 to prevent CRC. PPAR-γ activation can inhibit cell proliferation and migration, and promote CRC cell differentiation and apoptosis.^39–41^ Interestingly, serum PPAR-γ in CRC patients was significantly reduced and PPAR-γ showed a significant negative association with CRC in the current study. Reduced survival of patients with poor CRC is associated with down-regulated PPAR-γ expression,^42^ and functional loss-of-function mutations in PPAR-γ are associated with human CRC.^43^ Therefore, clinical studies also show that PPAR-γ is an important target for preventing CRC. Moreover, CLA can partially prevent tumor formation through a PPAR-γ-dependent mechanism,^44^ which was consistent with the results of this study. Thus, CCFM683 can prevent CRC via the CLA-large intestinal PPAR-γ axis. PPAR-γ is an important target for CRC, and represents a basis on which supplements can be developed to prevent CRC.

The intestinal mucosal barrier can protect the intestine from the invasion of toxins and pathogens. A decrease in MUC2 will increase the susceptibility to CRC,^45^ and clinical research has also found that a decrease in MUC2 will promote the occurrence of CRC,^46^ indicating that MUC2 is a potential target for CRC. Orally administered *L. casei* DSM 20011 can significantly up-regulate MUC2 concentration and prevent CRC,^47^ which was consistent with the current study. Tight connections between colonic epithelial cells can connect epithelial cells, regulate epithelial cell polarity, block microbial invasion, and inhibit the occurrence of CRC.^48^ *B. longum* subsp. *infantis* can inhibit the proliferation of CRC cells by regulating the expression of Occludin and Claudin-1^8^. In addition, PPAR-γ has been reported to up-regulate ZO-1 and Claudin-1 and regulate inflammation,^49,50^ while the current study showed similar results. In CRC patients, the proliferation of cancer cells in the colorectal tissue increases, and apoptosis is suppressed.^51^ In this study, treatments with CCFM683 and CCFM683Δ*bbi:bbi* promoted apoptosis of colonic epithelial cells, while CCFM683Δ*bbi* or CCFM683+GW9662 treatment had no significant effect. *B. bifidum* treatment can promote the apoptosis of colonic epithelial cells in CRC mice,^7^ which was consistent with the results of this study. However, the down-regulation of PPAR-γ expression promotes the overexpression of Bcl-2, inhibits Bax, and inhibits the apoptosis of liver cancer cells,^52^ indicating that PPAR-γ may promote cancer cell apoptosis. Thus, CCFM683 could prevent CRC by up-regulating MUC2, Claudin-1, and ZO-1, and promoting tumor cell apoptosis via the CLA-PPAR-γ axis.

When inflammation occurs in the intestine, harmful microbes are recognized and presented by antigen-presenting cells, which then guide T cells to differentiate, secrete a variety of pro-inflammatory cytokines, and promote the occurrence of CRC.^53^ Orally administered *B. longum* subsp. *infantis* can significantly down-regulate IL-6, IL-1β, and TNF-α in the colons of CRC rats, preventing CRC,^9^ while our study also suggests that CCFM683 can prevent CRC by regulating cytokines. In addition, inflammation, bleeding, and other symptoms caused by CRC are related to the activation of the NF-κB signaling pathway, and PPAR-γ is an important target for regulating this pathway.^54^ Therefore, we analyzed the effect of CCFM683 and PPAR-γ inhibitors on the NF-κB signaling pathway. Compared with PBS, treatments with CCFM683 and CCFM683Δ*bbi:bbi* significantly inhibited the NF-κB signaling pathway, while CCFM683Δ*bbi* or CCFM683+GW9662 treatment showed an in-significant effect. Orally administered *B. longum* can inhibit the NF-κB signaling pathway in the colons of CRC rats, down-regulate IL-1β and IL-6, and prevent CRC,^55^ indicating that overactivation of NF-κB leads to the expression of pro-inflammatory cytokines, promoting the occurrence of CRC. In addition, CLA can regulate CRC cells by inhibiting the NF-κB pathway, and activating PPAR-γ can inhibit the NF-κB signaling pathway,^56^ inhibiting the proliferation of CRC cells.^57^ Therefore, CCFM683 can prevent CRC by regulating the NF-κB signaling pathway and cytokines via the CLA-PPAR-γ axis. Inhibition of the NF-κB signaling pathway is an important strategy for the future prevention of CRC.

Gut microbiota imbalance can lead to changes in its composition and function, triggering chronic inflammation and increasing the risk of CRC.^4^ Gut microbiota played an important role in the prevention of CRC for CCFM683 using pseudogerm-free mice and FMT. It’s worth noting that there was a significant difference in the β-diversity of gut microbiota between the FMT-CCFM683Δ*bbi* group and the PBS group. FMT-CCFM683Δ*bbi* did not significantly prevent CRC. Therefore, we speculated that CCFM683Δ*bbi* can significantly alter the gut microbiota treated by PBS, but this change is not sufficient to prevent CRC. Metagenomic analysis showed that FMT-CCFM683 treatment significantly up-regulated the relative abundance of *O. splanchnicus*, while FMT-CCFM683Δ*bbi* showed no effect. Moreover, studies indicate a strong significant negative correlation between *O. splanchnicus* and the severity of CRC. *O. splanchnicus* can induce Th17 cell differentiation in both conventional and germ-free mice and has a certain protective effect against intestinal inflammation,^58^ which was consistent with the current study. Based on this, we speculated that *O. splanchnicus* may be a potential bacterium to prevent CRC. The cell-free supernatants of *O. splanchnicus* can promote apoptosis of CRC cells, induce anti-proliferative activity, and reduce the formation of CRC in mice.^13^ In the present study, orally administered *O. splanchnicus* can significantly prevent CRC. It has shown that *O. splanchnicus* can up-regulate ZO-1, MUC2, and goblet cells to prevent the intestinal mechanical barrier *in vitro* ,^59^ while the same results were found in the current study *in vivo*. In an *in vitro* study, it has been shown that *O. splanchnicus* can induce immune cells to produce IL-10, which may play an anti-inflammatory role in the intestinal epithelium.^60^ In addition, it has been found that *O. splanchnicus* can metabolize butyric acid, promote the differentiation of Treg cells, induce the secretion of IL-10, and inhibit intestinal inflammation in mice.^61^ In the current study, *O. splanchnicus* regulated IL-10. Interestingly, *O*. *splanchnicus* increased the content of butyric acid in CRC mice. Both cellular and animal studies have shown that butyric acid has a regulatory effect on CRC.^62,63^ Therefore, butyric acid may be a potential substance to prevent CRC. *O. splanchnicus* treatment significantly increased the relative abundances of *Bifidobacterium* and *Faecalibaculum*, which were significantly negatively associated with CRC, similar to previous reports.^6,64^ This study showed that orally administered *Bifidobacterium* can increase the relative abundance of *O. splanchnicus*, while treatment with *O. splanchnicus* can increase the abundance of *Bifidobacterium*. Therefore, we speculated that *Bifidobacterium* and *O. splanchnicus* might have a trophic mutualism relationship, which needs further investigation.

## 5. Conclusion

In conclusion, compared with healthy volunteers, the content of CLA, butyric acid, and *Bifidobacterium* in the intestine of CRC patients was reduced, and the content of PPAR-γ and anti-inflammatory cytokines in the serum was reduced. However, supplementation of CCFM683 can prevent CRC by producing CLA in the intestine to regulate cytokines, inhibit the NF-κB signaling pathway, up-regulate intestinal TJ proteins and MUC2, and regulate gut microbiota, and all these effects were PPAR-γ dependent. Based on FMT, the potential bacterium (*O. splanchnicus*) for preventing CRC was screened which showed that *O*. *splanchnicus* prevented CRC by regulating intestinal cytokines, up-regulating TJ proteins and MUC2, and regulating gut microbiota (Figure S12). These results contribute to the understanding of the mode of *Bifidobacterium* in preventing CRC and regulating immune-related diseases and have guiding significance for clinical trials of *Bifidobacterium* and the development of CRC functional products.

## Supplementary Material

Supplemental Material

## Data Availability

The 16S rRNA gene sequencing data of feces in people (CRC patients and healthy volunteers) and Apc^Min/+^ mice generated in this study are deposited in NCBI Sequence Read Archive (SRA) under project ID PRJNA1034270 and PRJNA1034269. The shotgun metagenomic sequencing data of feces and transcriptome sequencing data of colon in Apc^Min/+^ mice generated in this study are deposited in NCBI SRA database under project ID PRJNA1034267 and PRJNA1034271.
