# Supplementary material for: Bifidobacterium inhibits the progression of colorectal tumorigenesis in mice through fatty acid isomerization and gut microbiota modulation
Source: Gut Microbes. 2025 Feb 9;17(1):2464945. doi: 10.1080/19490976.2025.2464945 (PMC11812354; doi:10.1080/19490976.2025.2464945)
Supplement: Supplemental Material [file KGMI_A_2464945_SM6624.zip › Supplementary Material-figures captions.docx]

**FIGURE CAPTIONS**

**Figure S1.** **The analysis of differences between CRC patients and healthy volunteers.**

(A-D) The concentration of fecal SCFAs.

*n* = 40 people per group. Independent-samples test-test. ****: p < 0.0001, ***: p < 0.001, **: p < 0.01.

**Figure S2. Effects of CLA-producing *Bifidobacterium* on CRC mice.**

Representative images of colon surveillance at week 16.

**Figure S3. Analysis of key metabolite of CCFM683 in relieving CRC.**

(A) Representative images of colon surveillance at week 16. The CLA concentration in the liver (B) and serum (C).

*n* = 8 mice per group. One-way ANOVA followed by Tukey multiple-comparison test. The p-value showed the difference that all groups were compared to PBS groups. ****: p < 0.0001, ***: p < 0.001, **: p < 0.01.

**Figure S4. Effects of *bb*i gene on CRC.**

(A) Representative images of colon surveillance at week 16. The CLA concentration in the liver (B) and serum (C).

*n* = 8 mice per group. One-way ANOVA followed by Tukey multiple-comparison test. The p-value showed the difference that all groups were compared to PBS groups. ****: p < 0.0001, ***: p < 0.001, **: p < 0.01.

**Figure S5. Analysis of key receptor of CCFM683 in relieving CRC.**

Representative images of colon surveillance at week 16.

**Figure S6. Effects of CCFM683 on the intestinal mechanical barrier in CRC mice.**

(A) Immunohistochemical staining for Bax and Bcl-2. The mean optical density values of Bax (B) and Bcl-2 (C).

*n* = 8 mice per group. One-way ANOVA followed by Tukey multiple-comparison test. The p-value showed the difference that all groups were compared to PBS groups. ****: p < 0.0001, ***: p < 0.001, **: p < 0.01, *: p < 0.05.

**Figure S7. Effects of CCFM683 on the intestinal immune barrier in CRC mice.**

The concentration of IL-6 (A) and IFN-γ (B) in colonic tissue.

*n* = 8 mice per group. One-way ANOVA followed by Tukey multiple-comparison test. The p-value showed the difference that all groups were compared to PBS groups. ****: p < 0.0001, ***: p < 0.001, **: p < 0.01, *: p < 0.05.

**Figure S8. Effects of FMT on CRC mice.**

(A) Representative images of colon surveillance at week 16. The relative abundance of Bacteroidetes (B) and Firmicutes (C).

*n* = 6-8 mice per group. One-way ANOVA followed by Tukey multiple-comparison test. The p-value showed the difference that all groups were compared to PBS groups. ****: p < 0.0001, ***: p < 0.001, **: p < 0.01, *: p < 0.05.

**Figure S9. Effects of *O. splanchnicus* on CRC mice.**

Representative images of colon surveillance at week 16.

**Figure 10. Effects of *O. splanchnicus* on intestinal mechanical barrier and immune barrier in CRC mice.**

The concentration of IL-6 (A) and IFN-γ (B) in colonic tissue.

*n* = 8 mice per group. Independent-samples test-test. ****: p < 0.0001, ***: p < 0.001, **: p < 0.01.

**Figure 11. Effects of *O. splanchnicus* on SCFAs and gut microbiota in CRC mice.**

(A-E) The concentration of fecal SCFAs. The relative abundance of Verrucomicrobia (F), Firmicutes (G), Proteobacteria (H), and Bacteroidetes (I).

*n* = 8 mice per group. Independent-samples test-test. ****: p < 0.0001, ***: p < 0.001, **: p < 0.01.

**Fig.S12.** The schematic of the potential mechanism of CCFM683 in alleviating CRC
