# Supplementary figures and images for: Bifidobacterium inhibits the progression of colorectal tumorigenesis in mice through fatty acid isomerization and gut microbiota modulation
Source: Gut Microbes. 2025 Feb 9;17(1):2464945. doi: 10.1080/19490976.2025.2464945 (PMC11812354; doi:10.1080/19490976.2025.2464945)

Fig.S1A

**
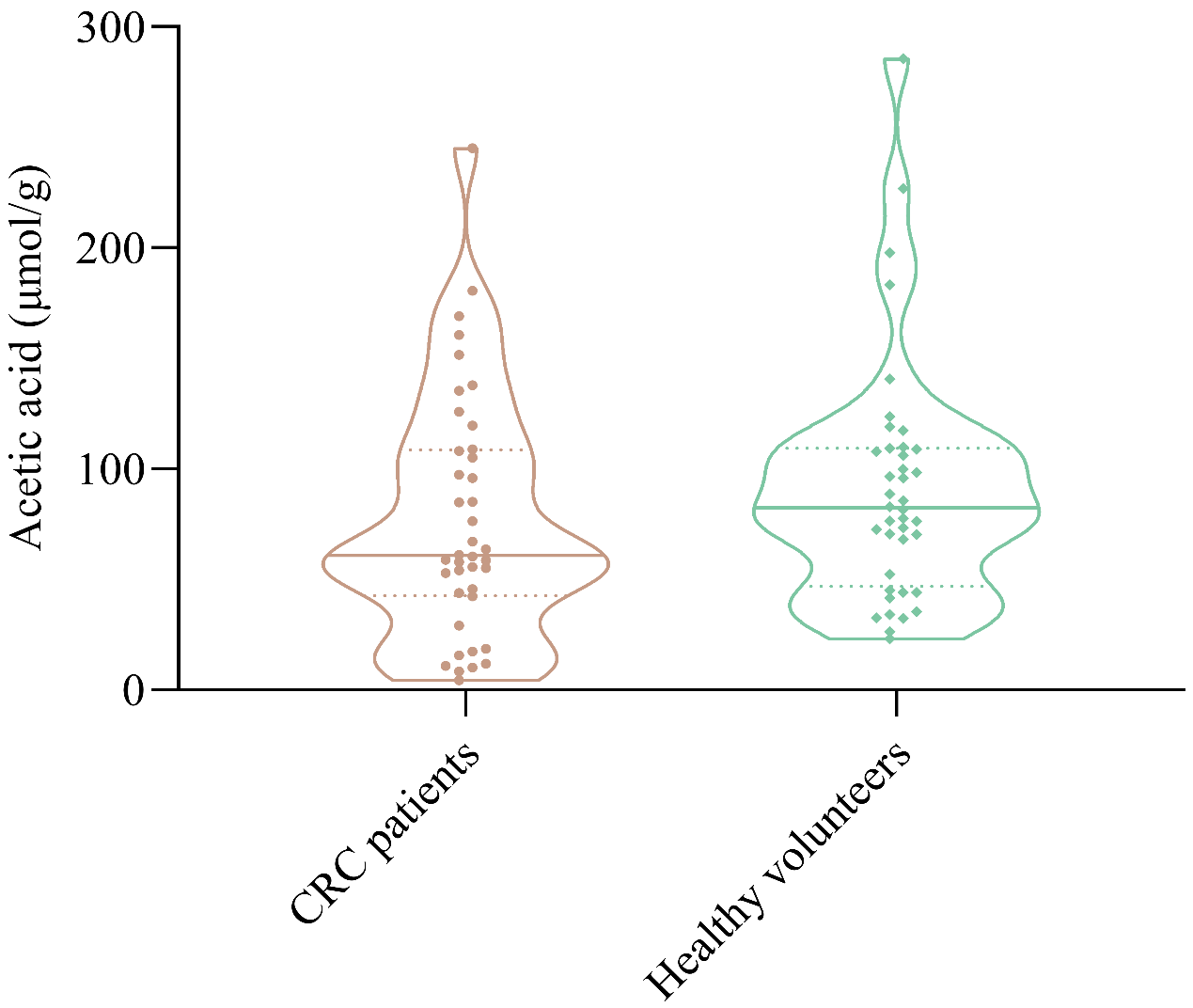
**

Fig.S1B


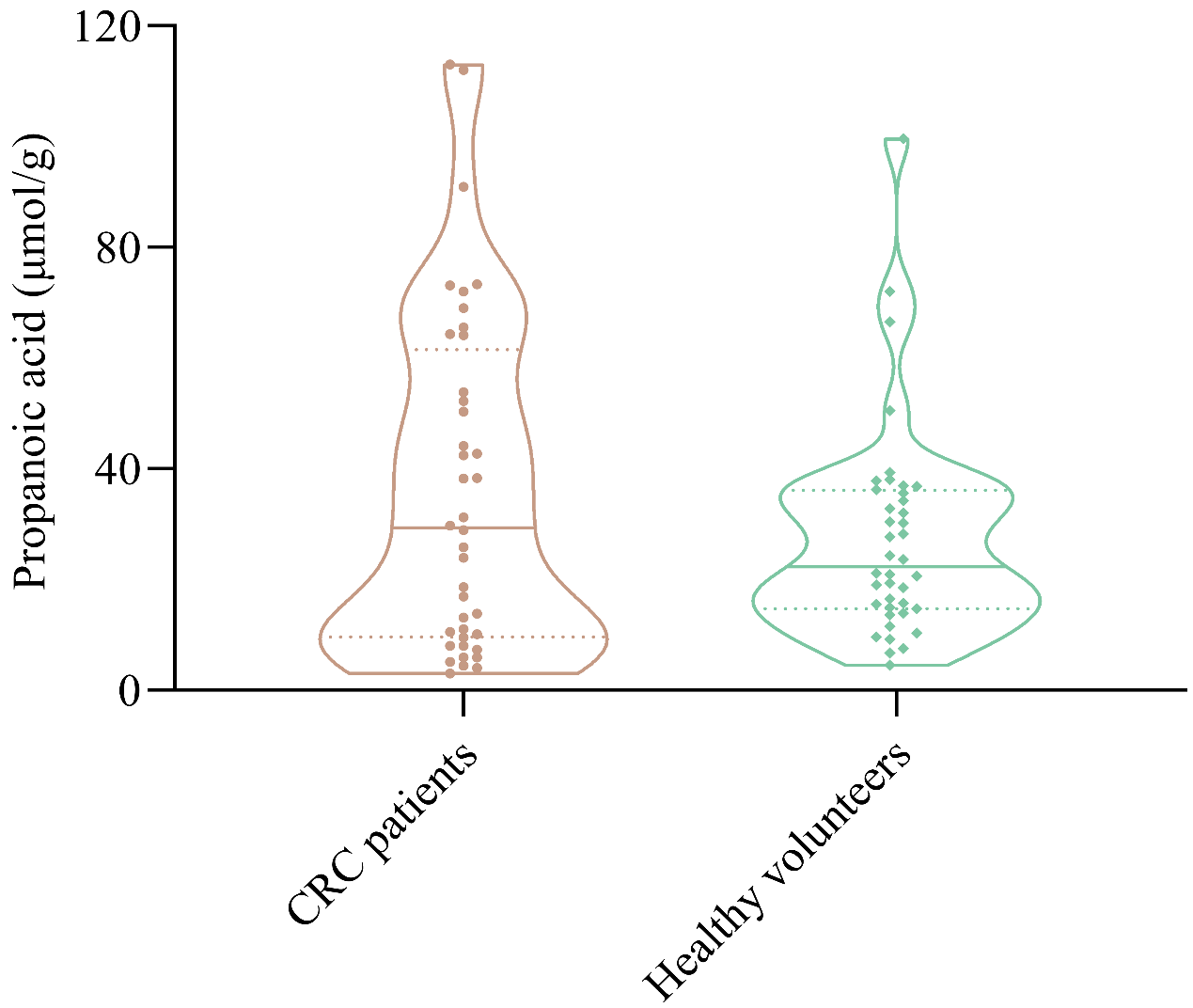


Fig.S1C


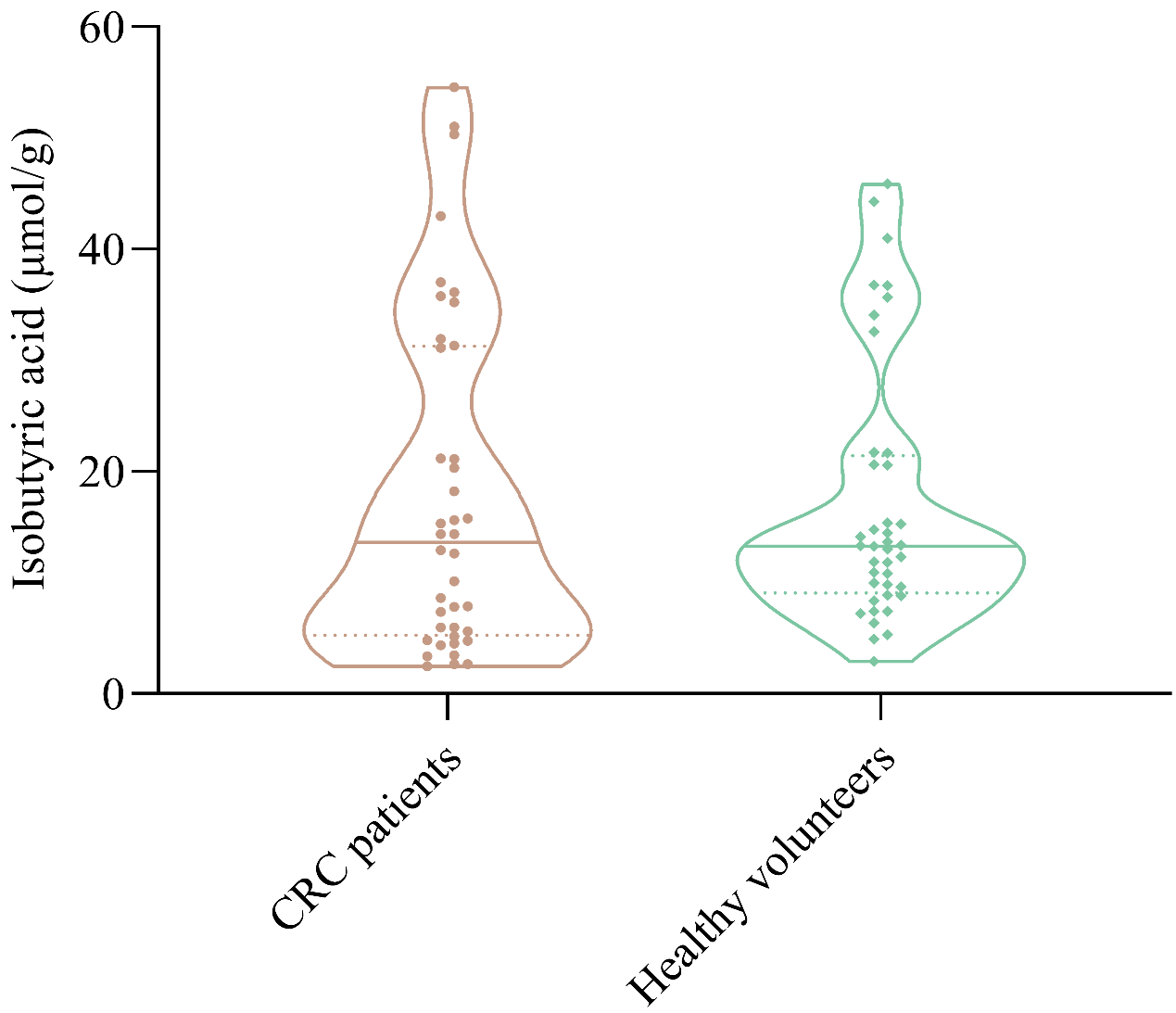


Fig.S1D


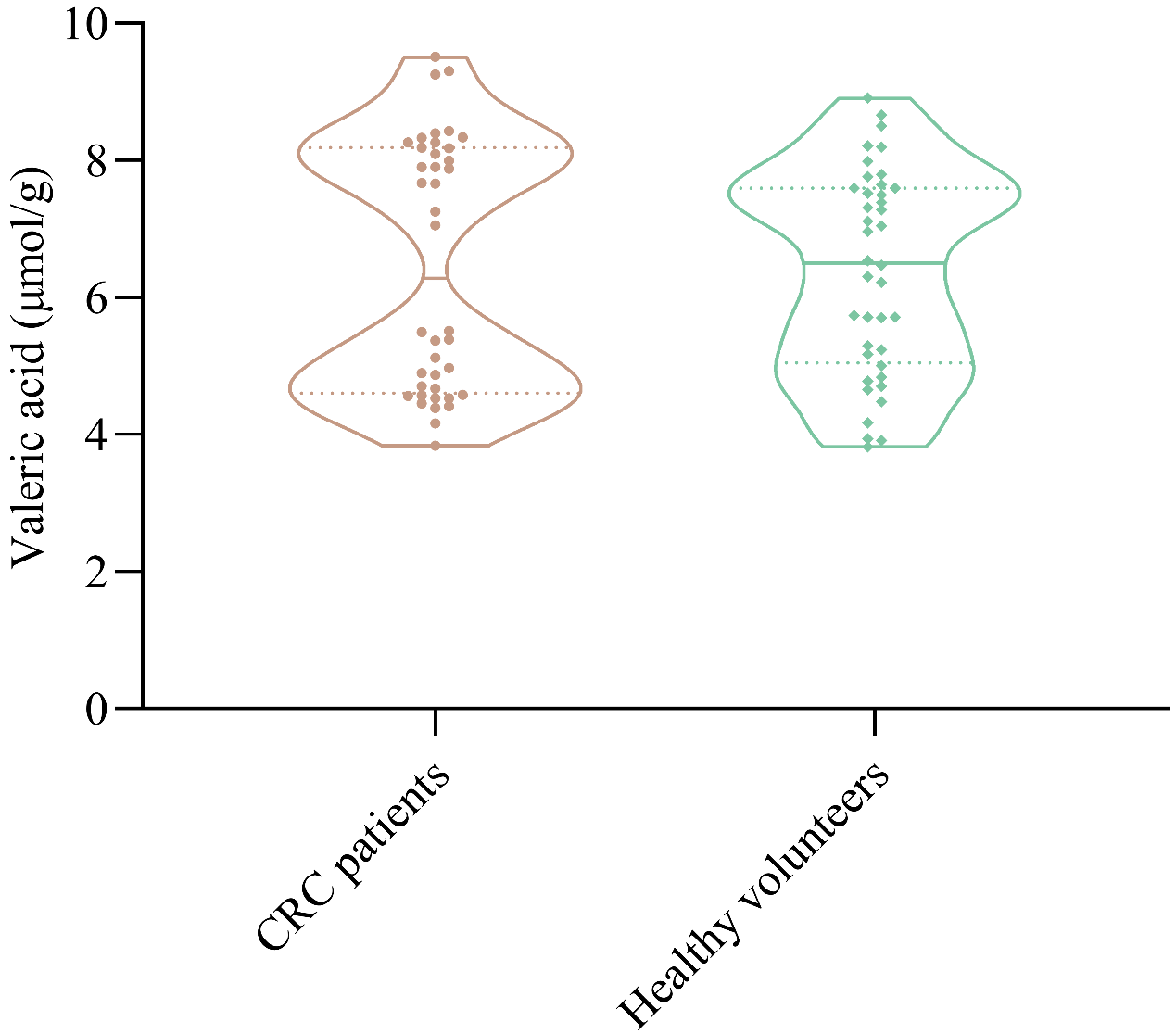


Fig.S2


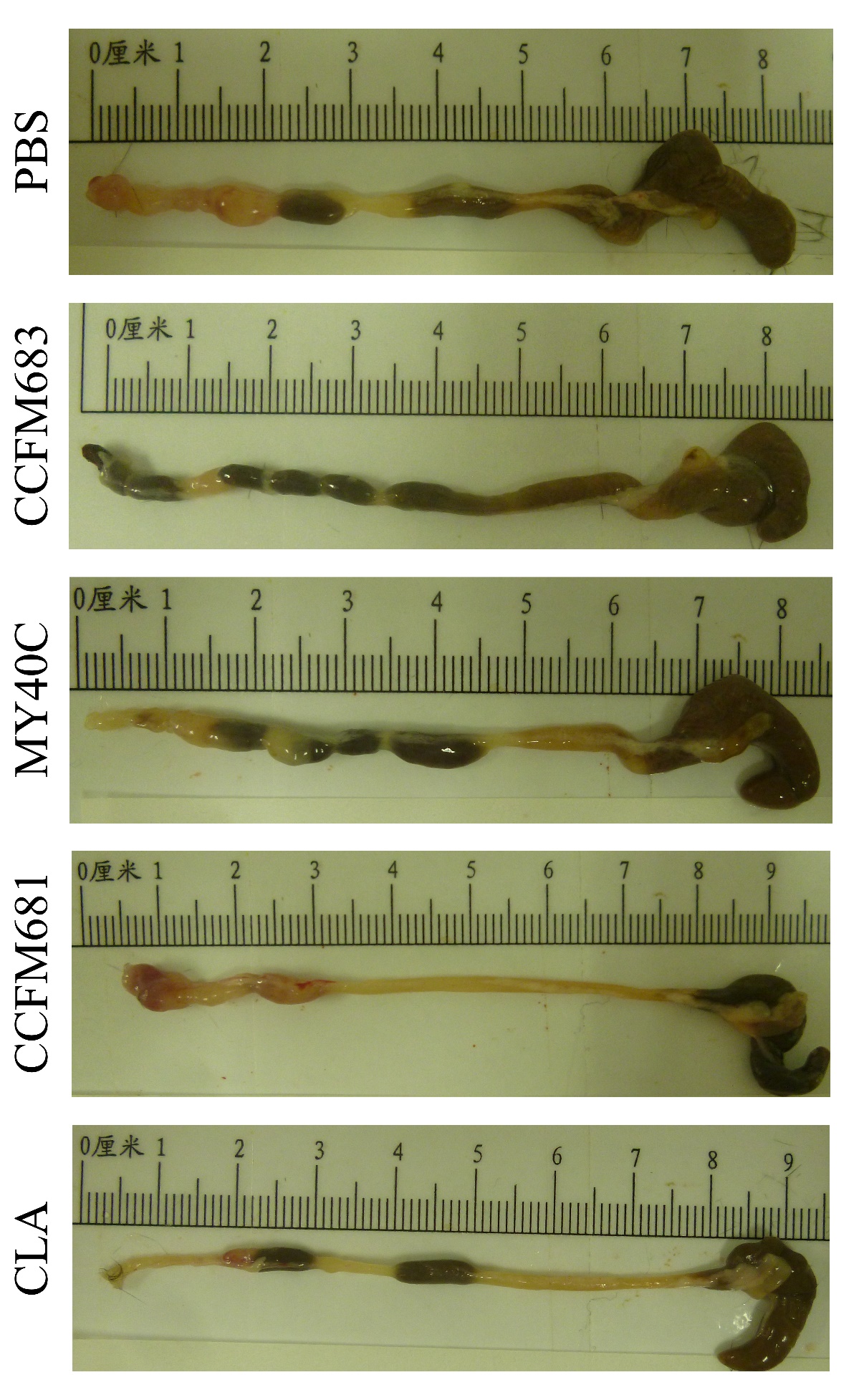


Fig.S3A


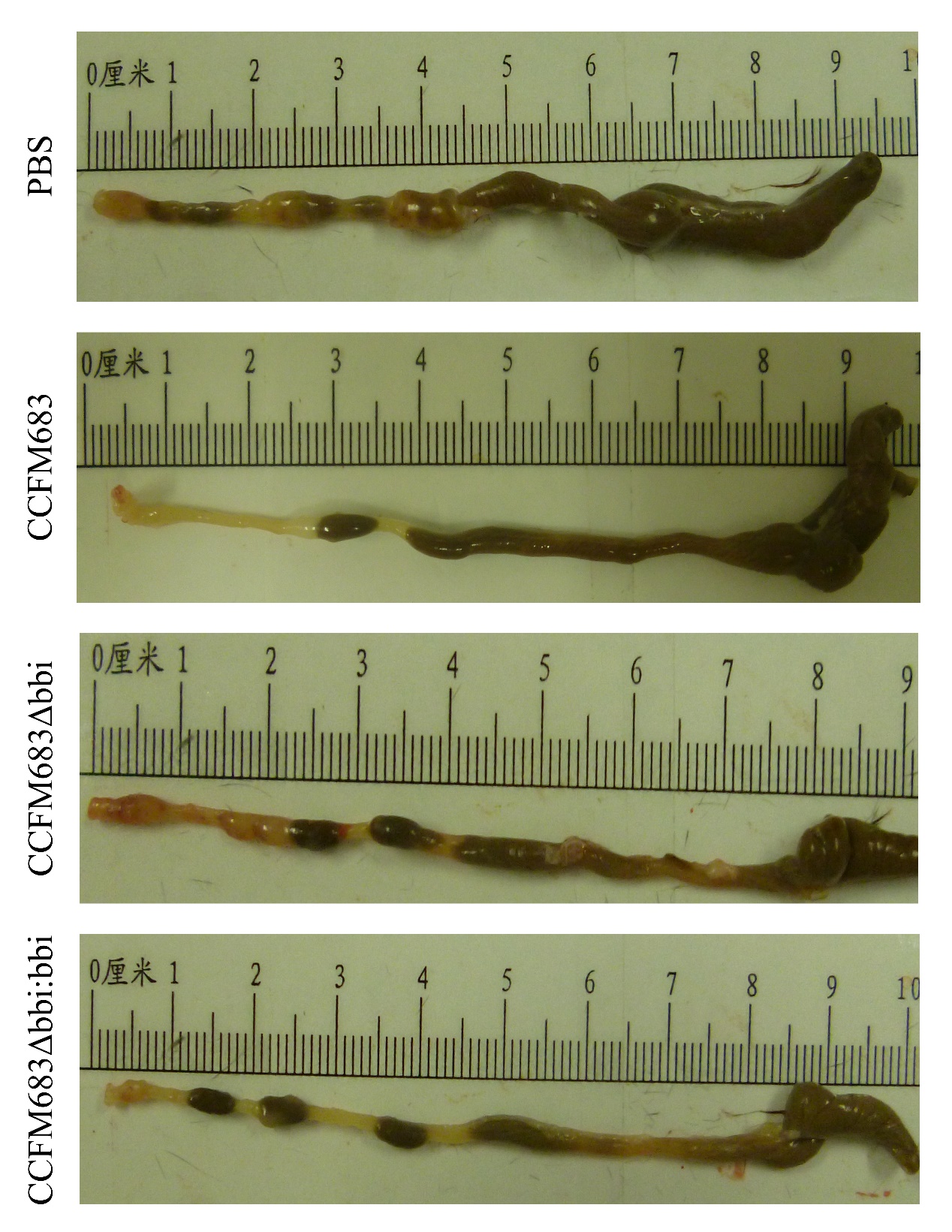


Fig.S3B


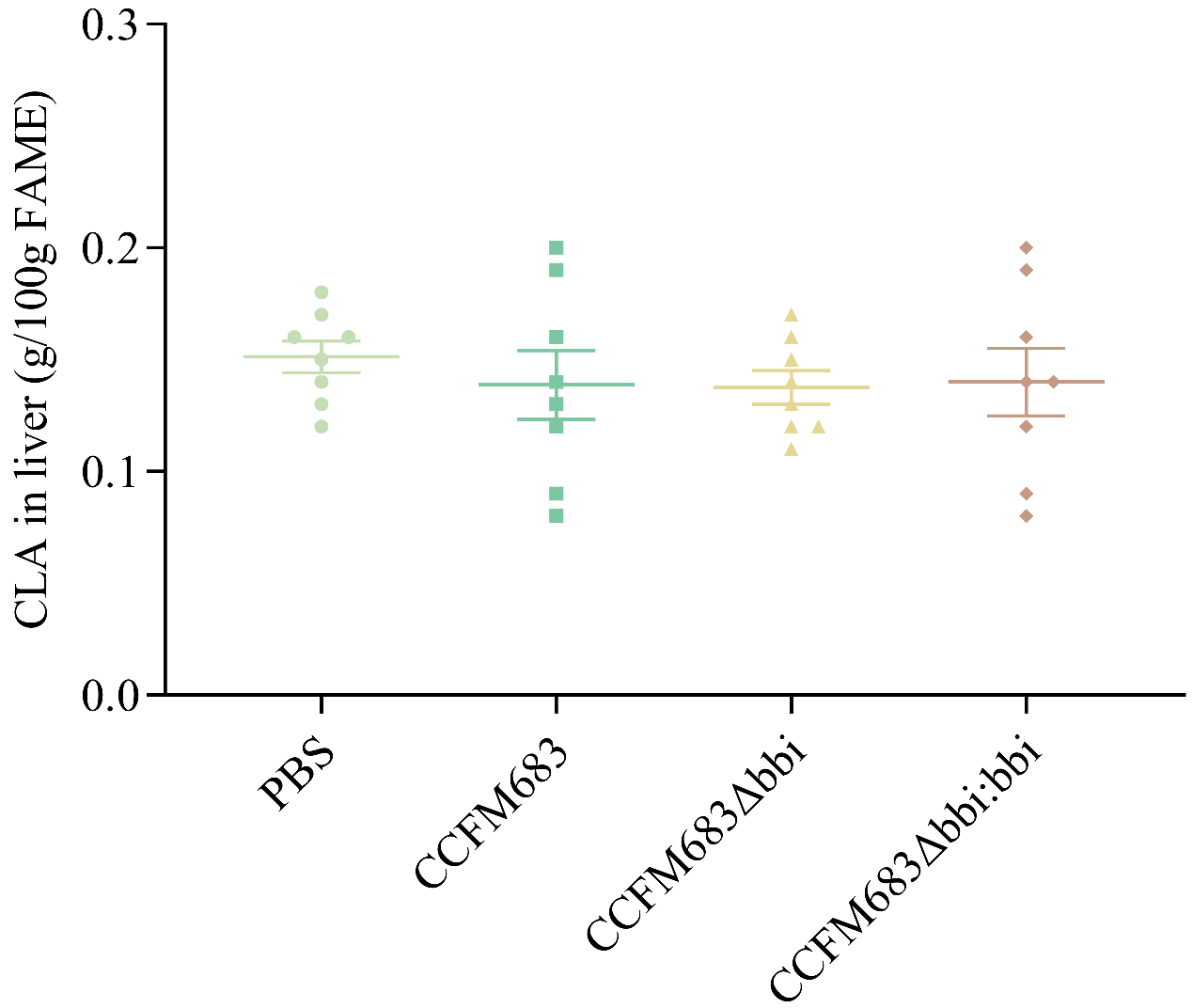


Fig.S3C


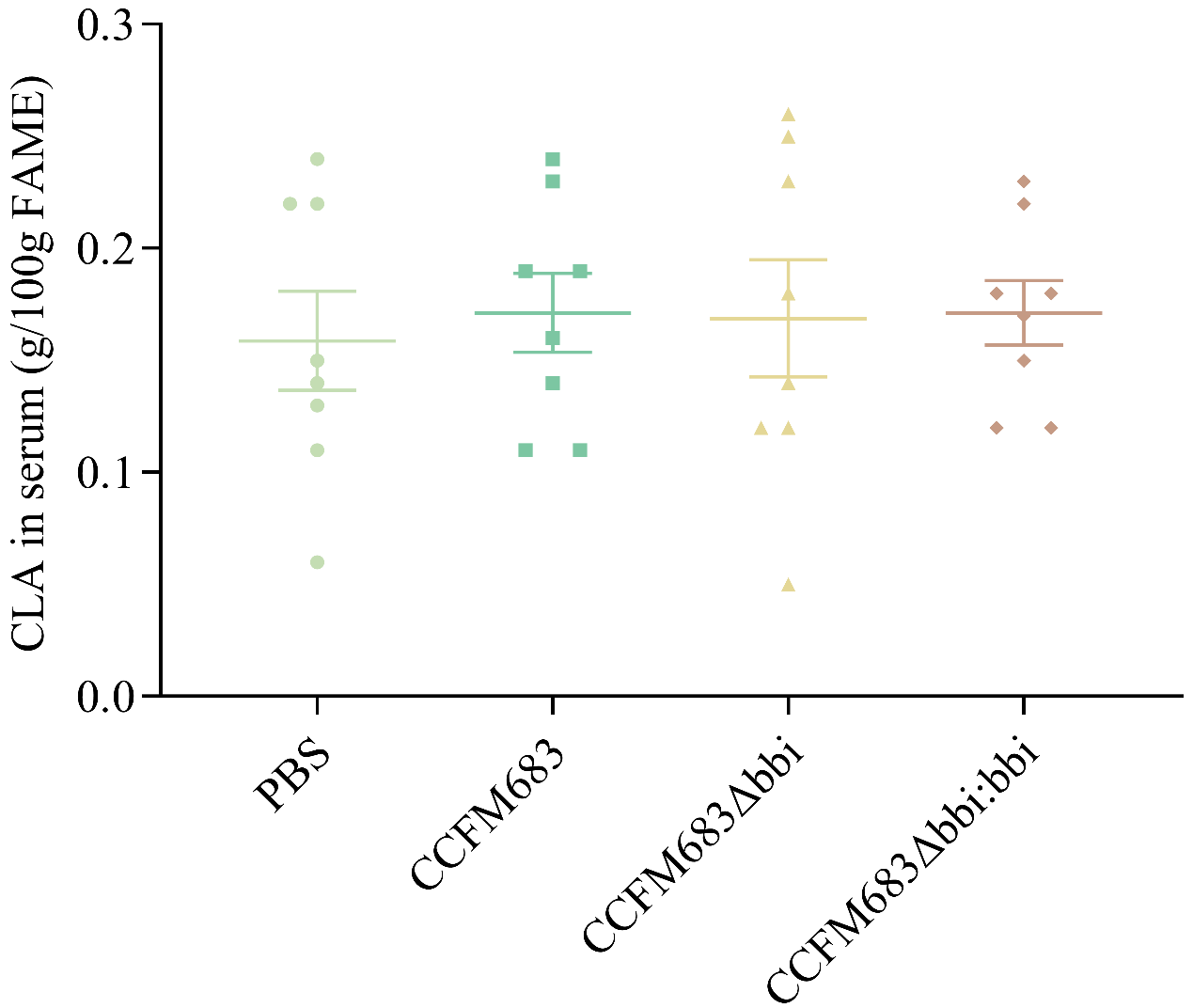

Supplement: Supplemental Material [file KGMI_A_2464945_SM6624.zip › supp s1-s3.docx]

Fig.S8A


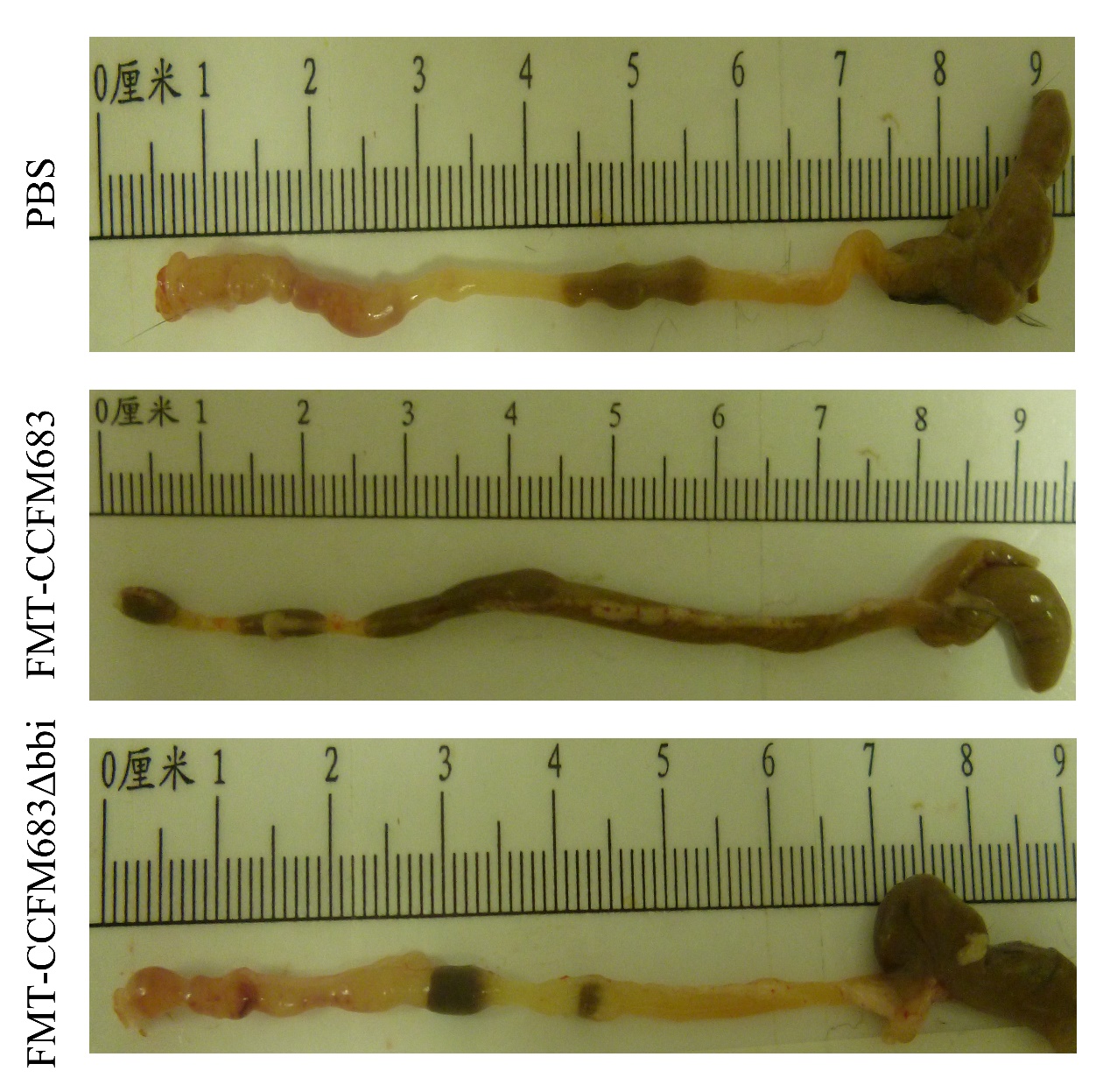


Fig.S8B


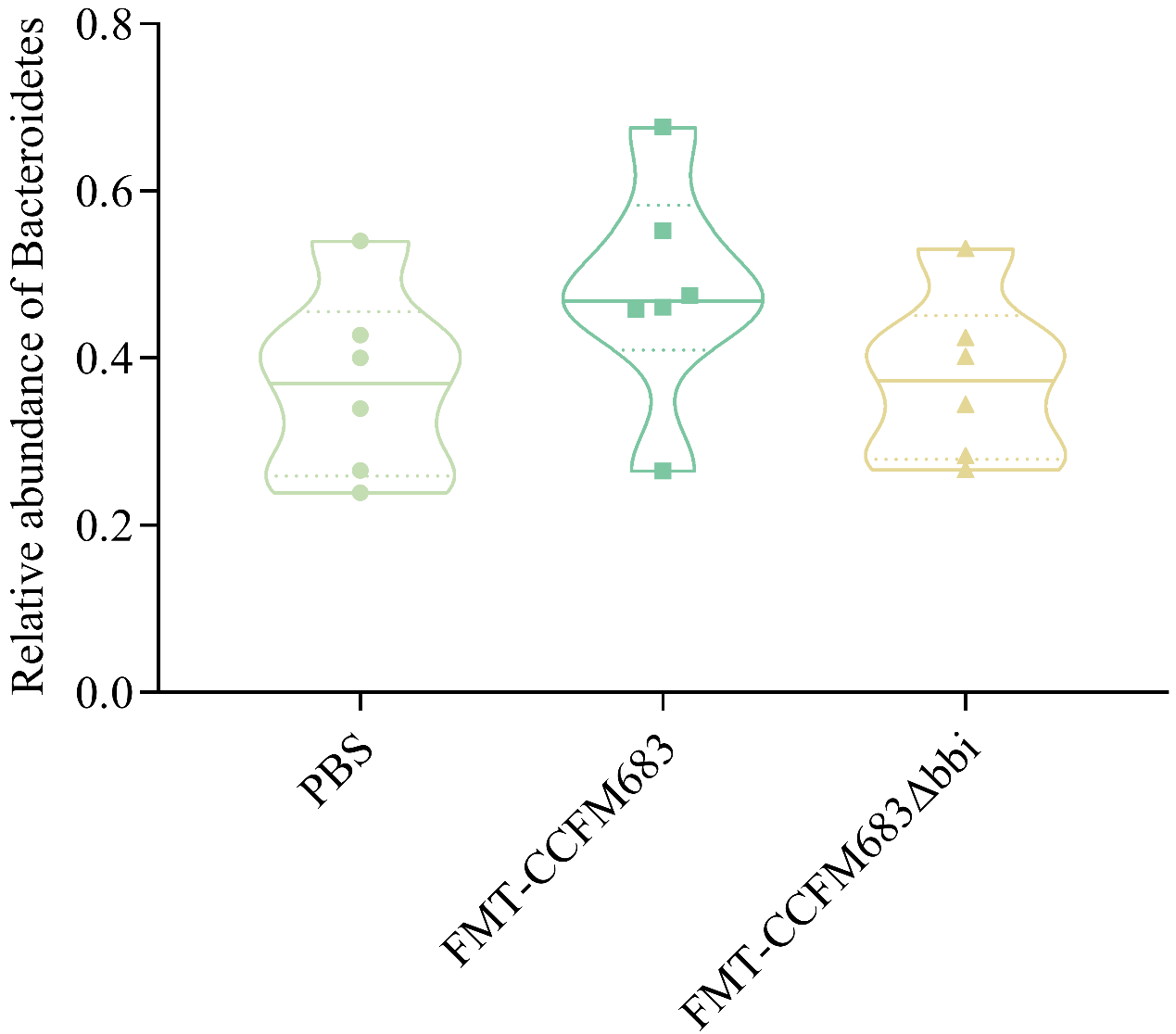


Fig.S8C


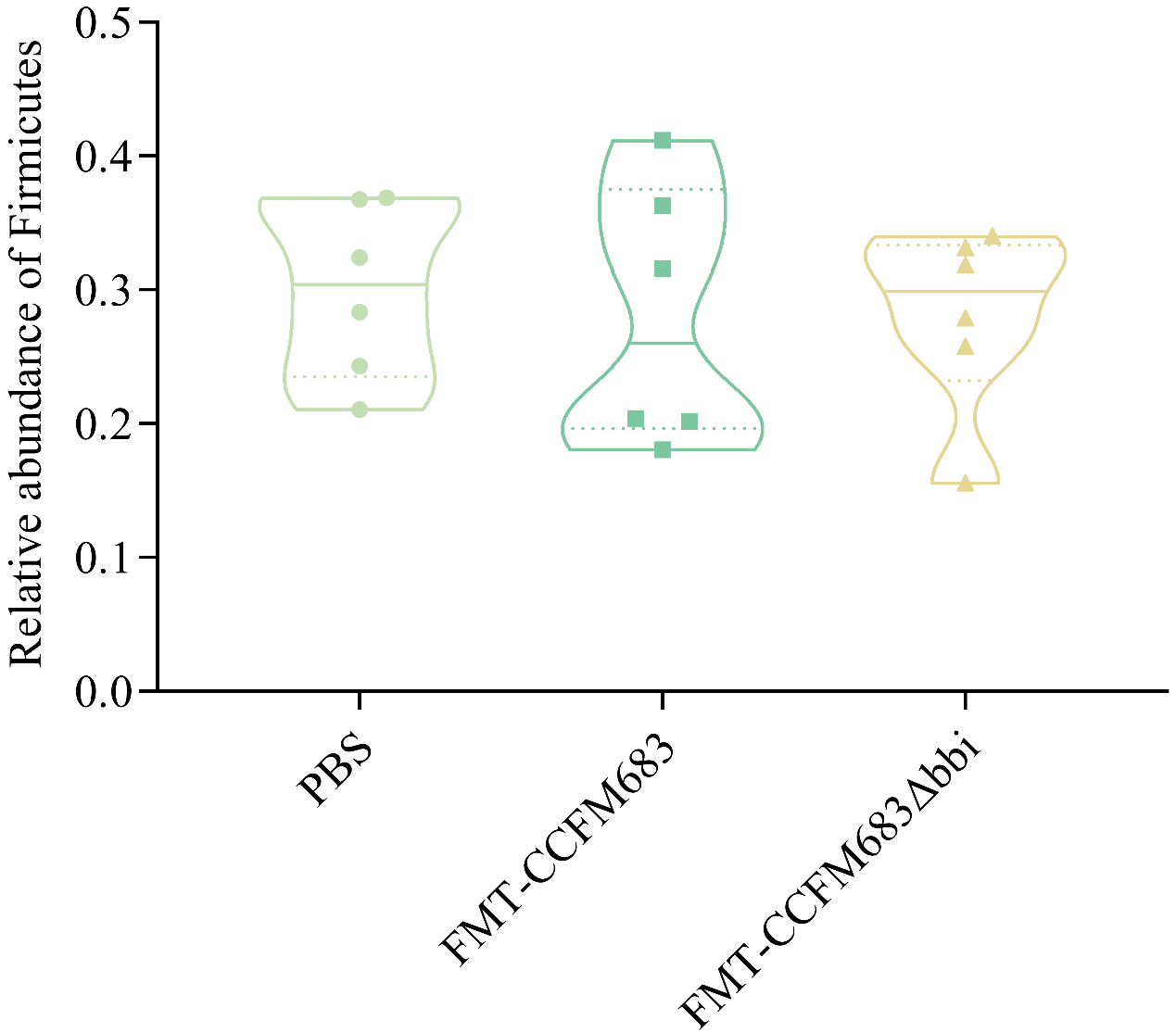


Fig.S9


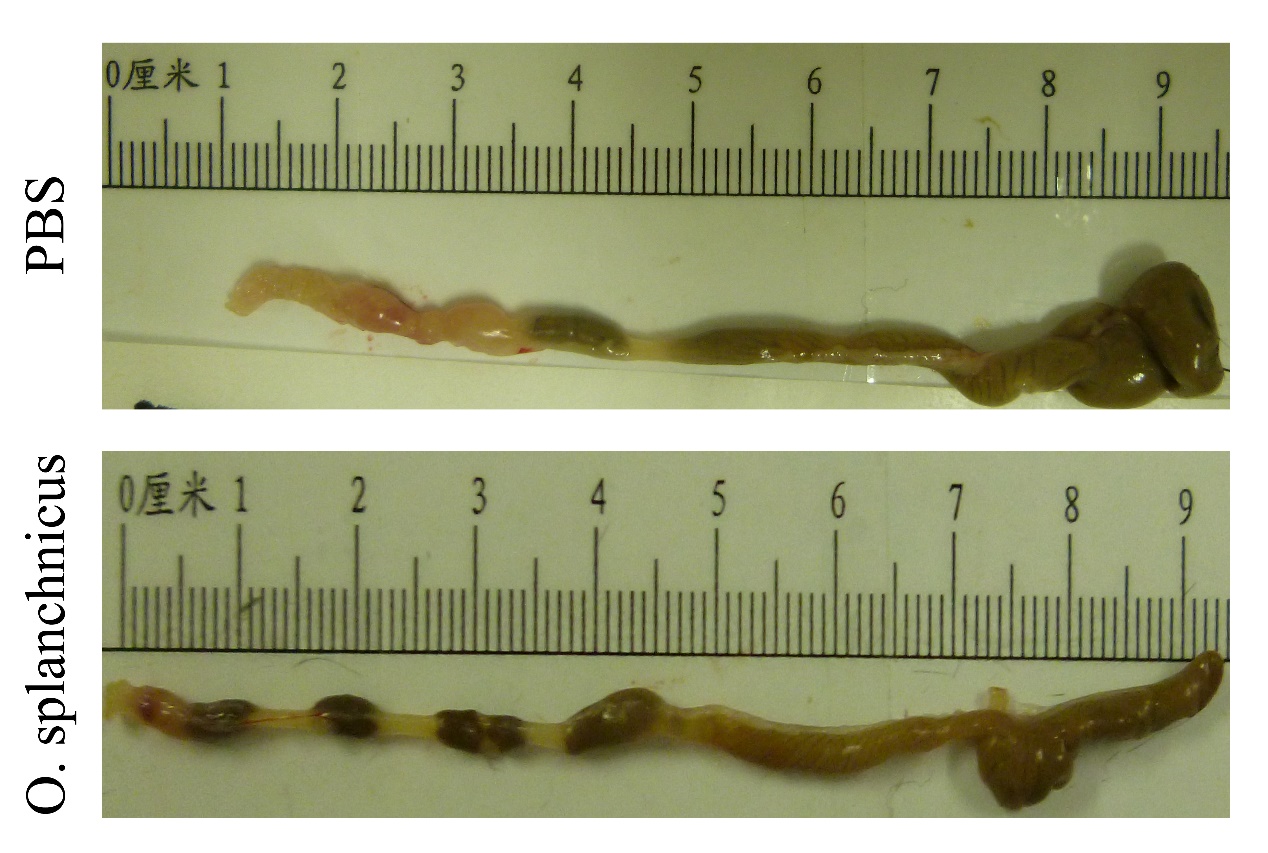


Fig.S10A
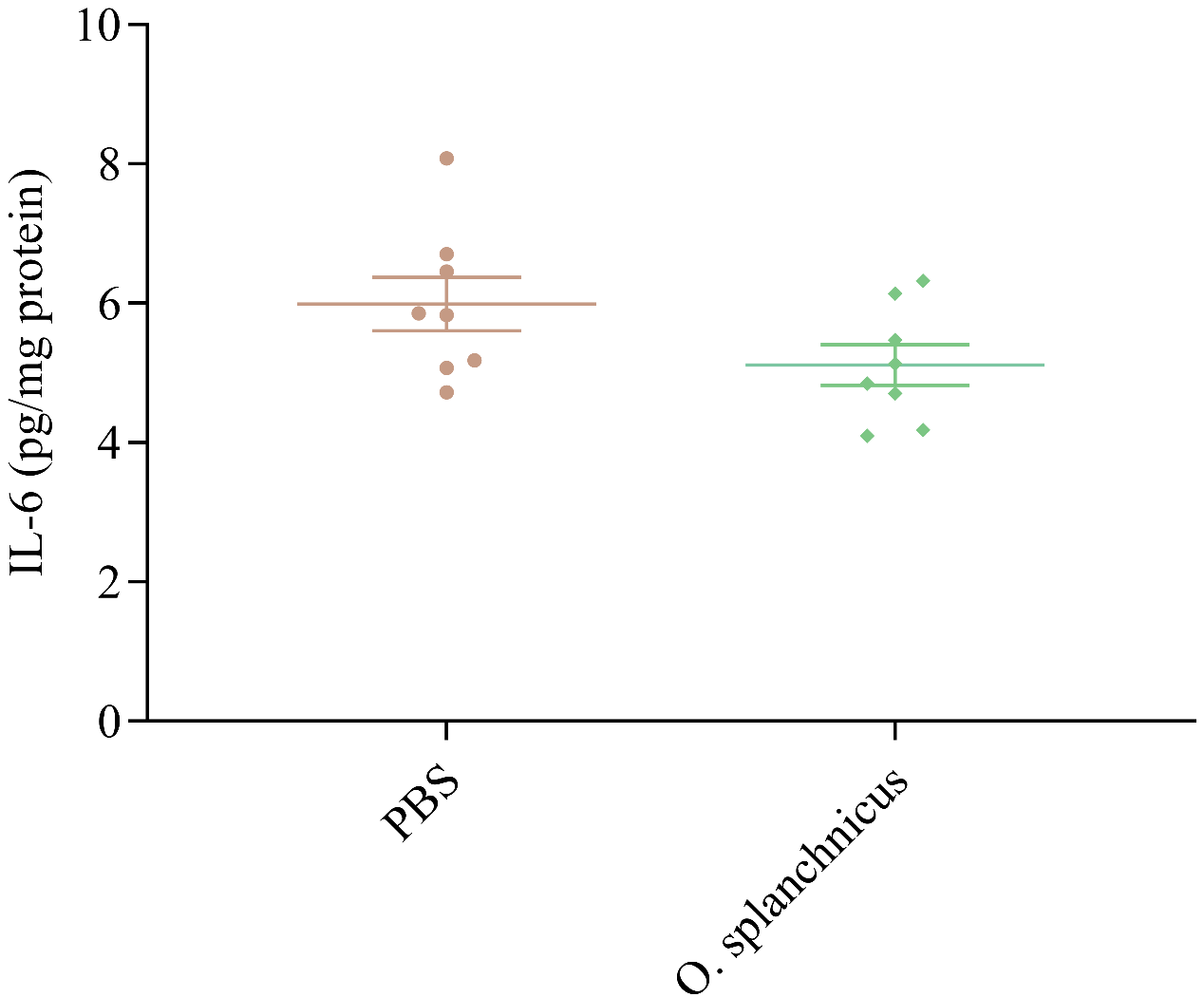


Fig.S10B


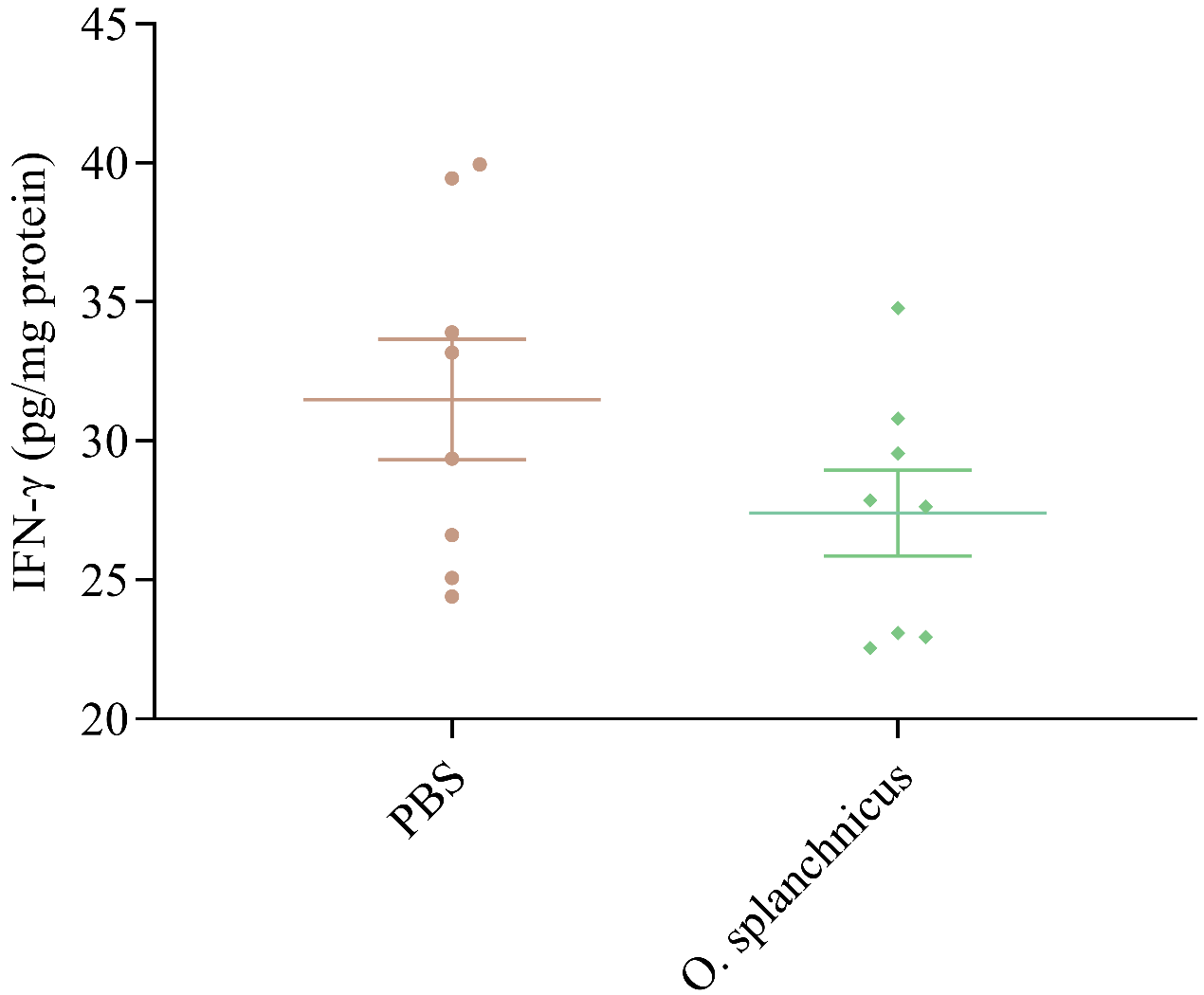


Fig.S11A


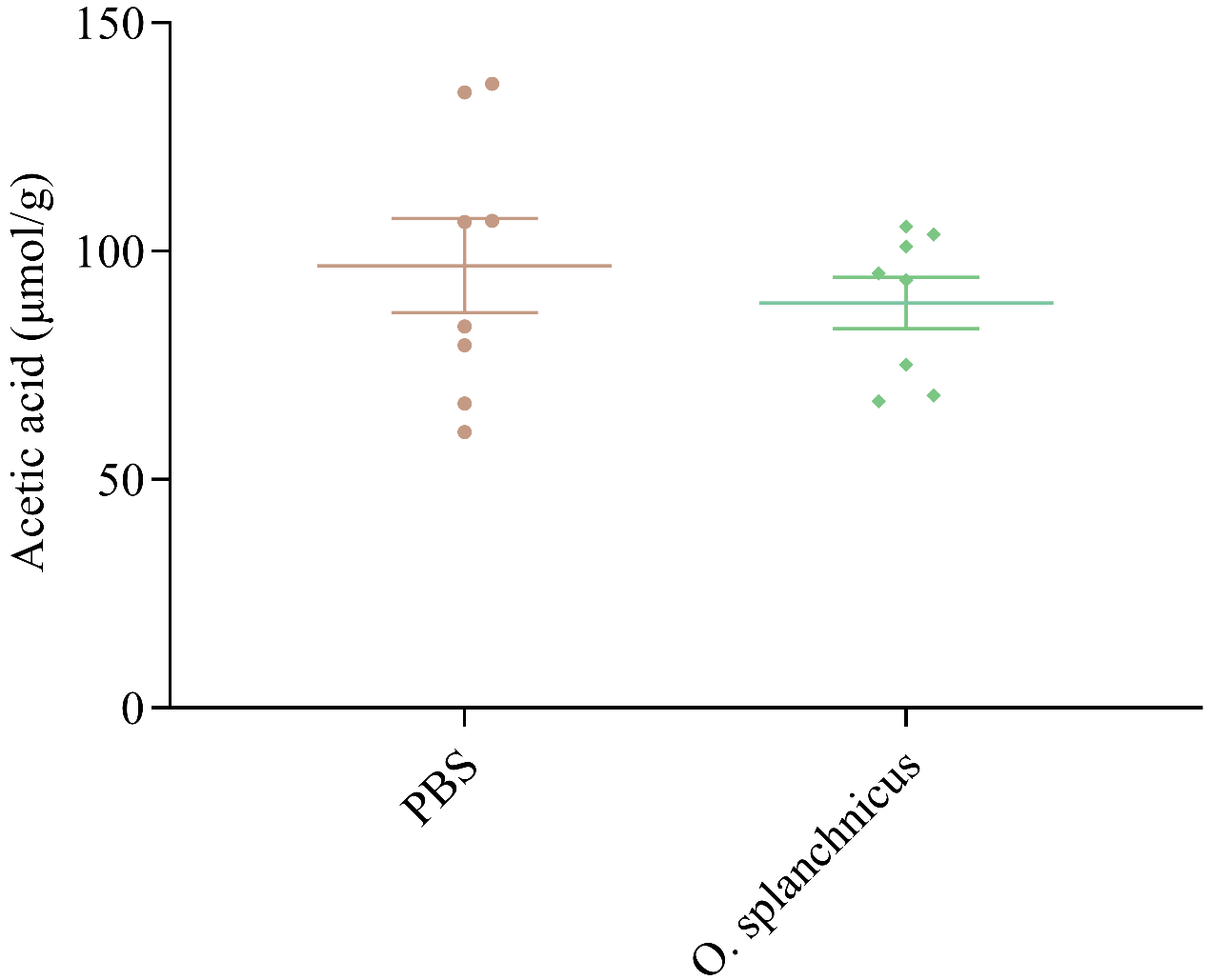


Fig.S11B


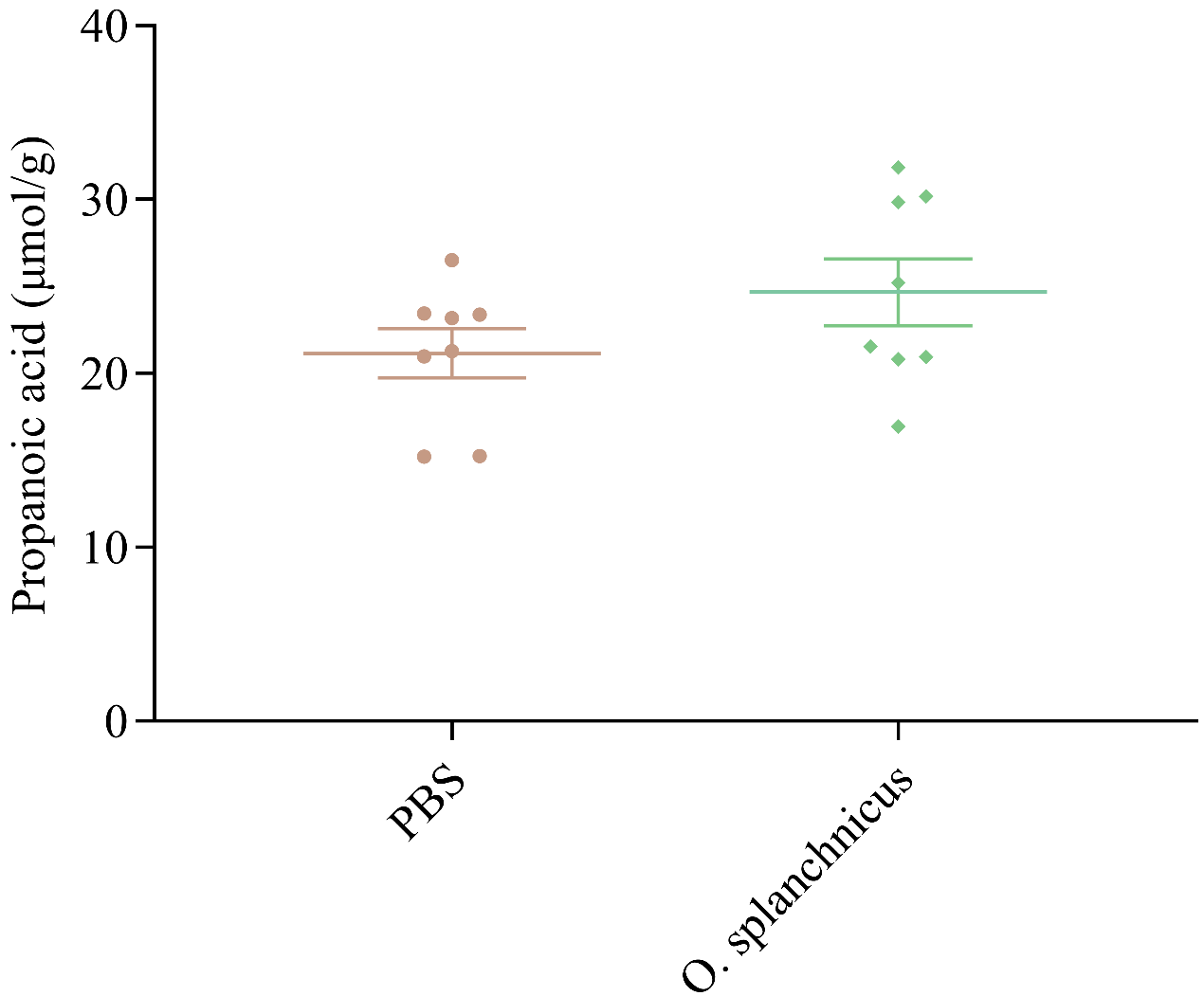


Fig.S11C


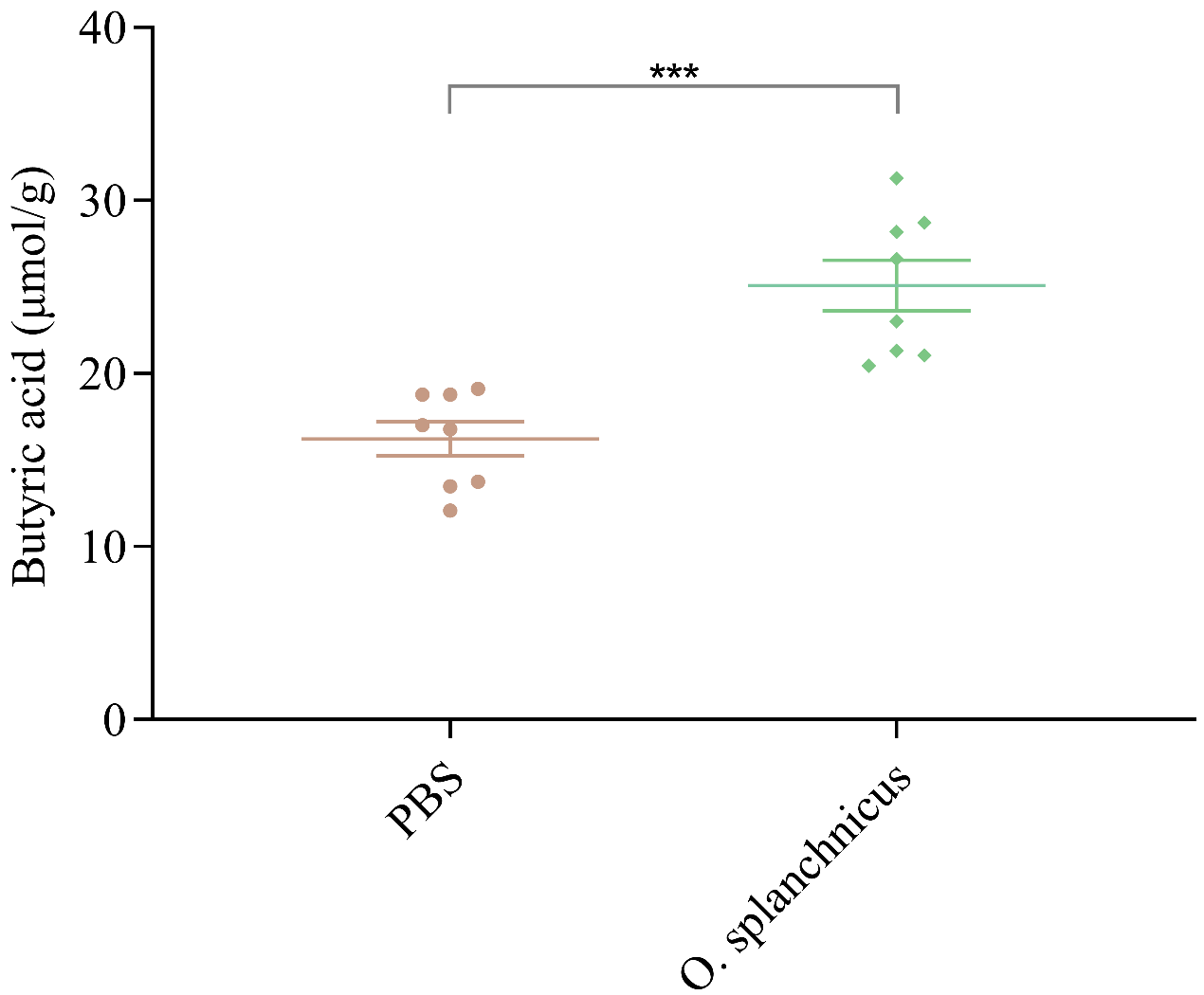


Fig.S11D


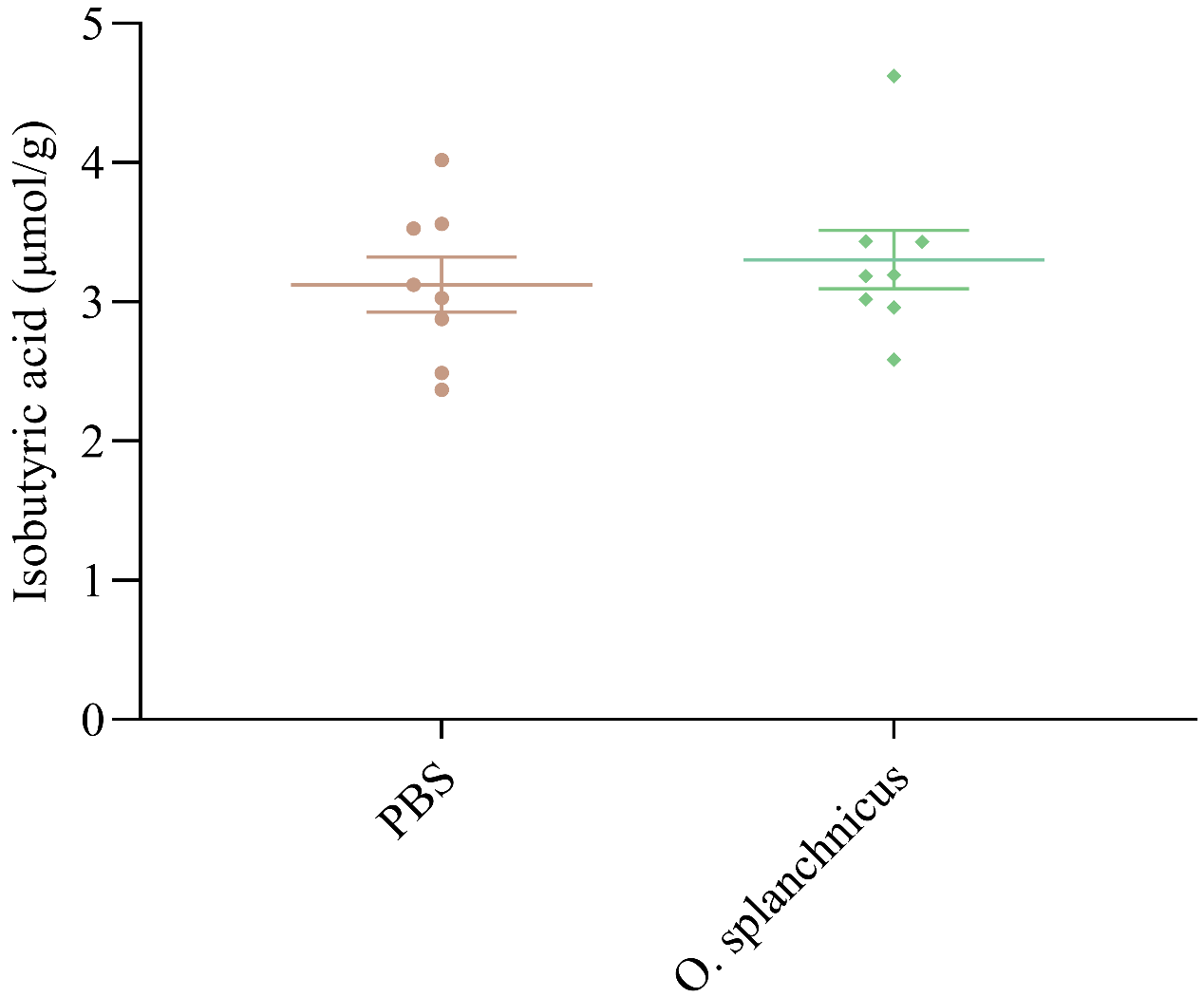


Fig.S11E


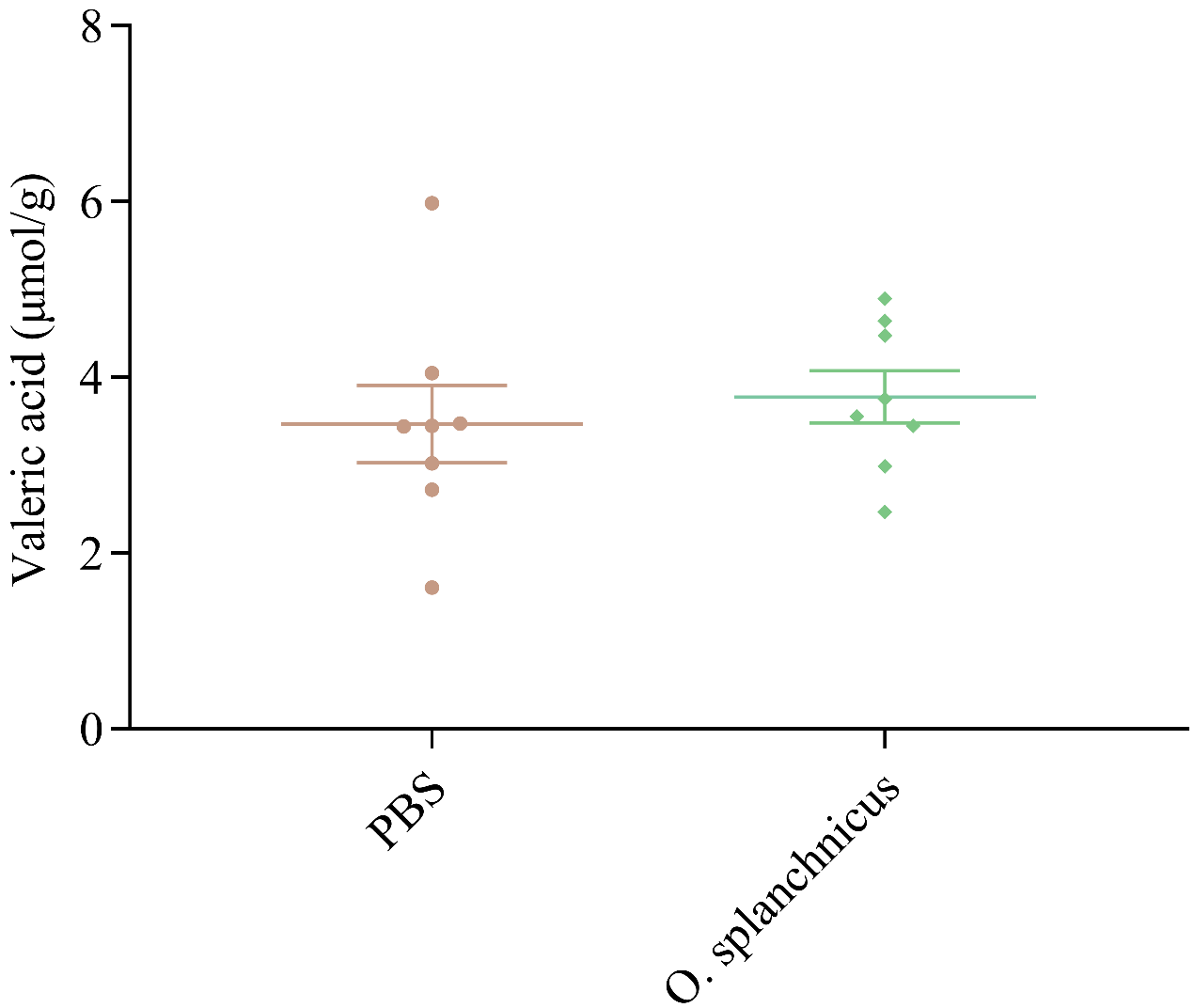


Fig.S11F


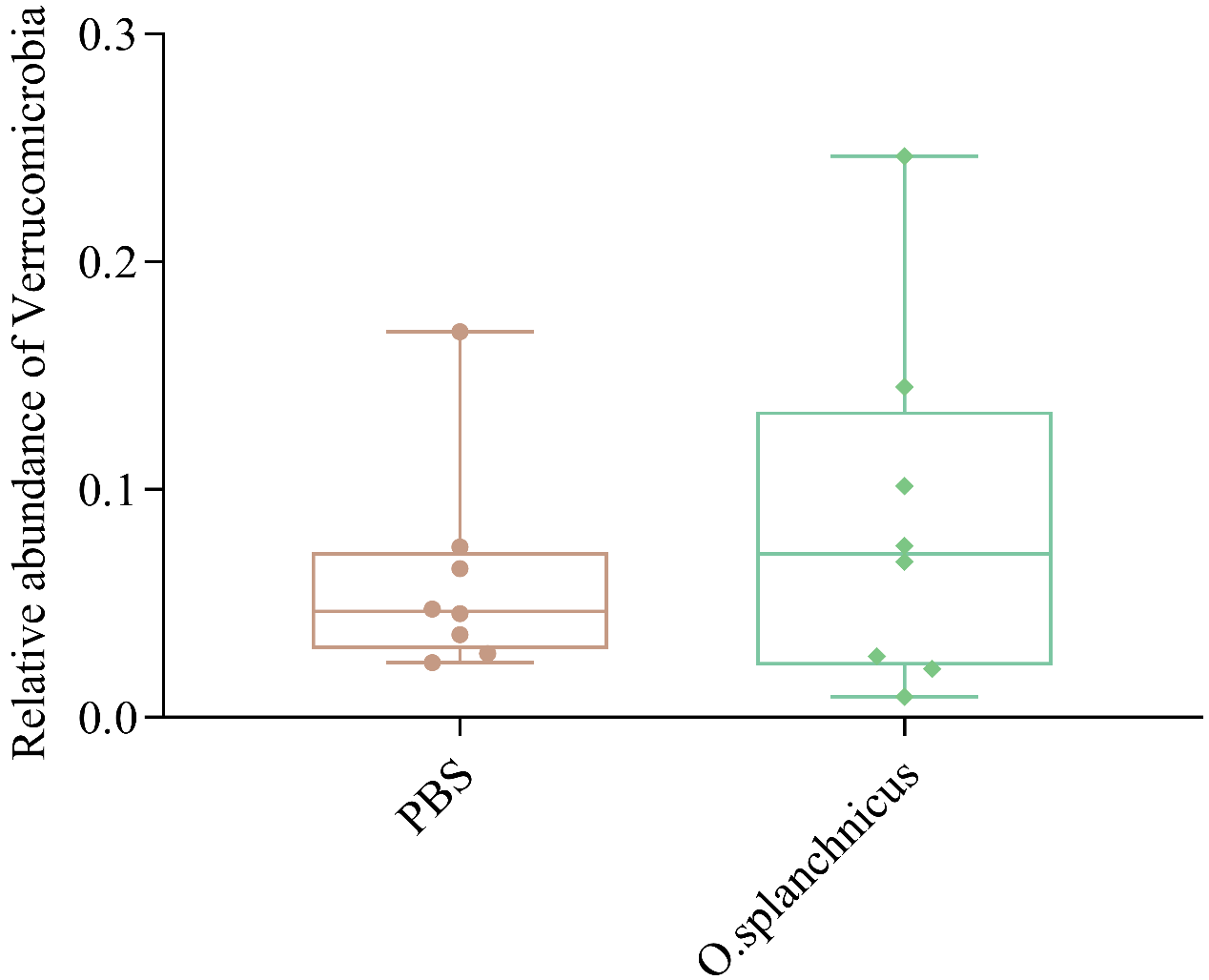


Fig.S11G


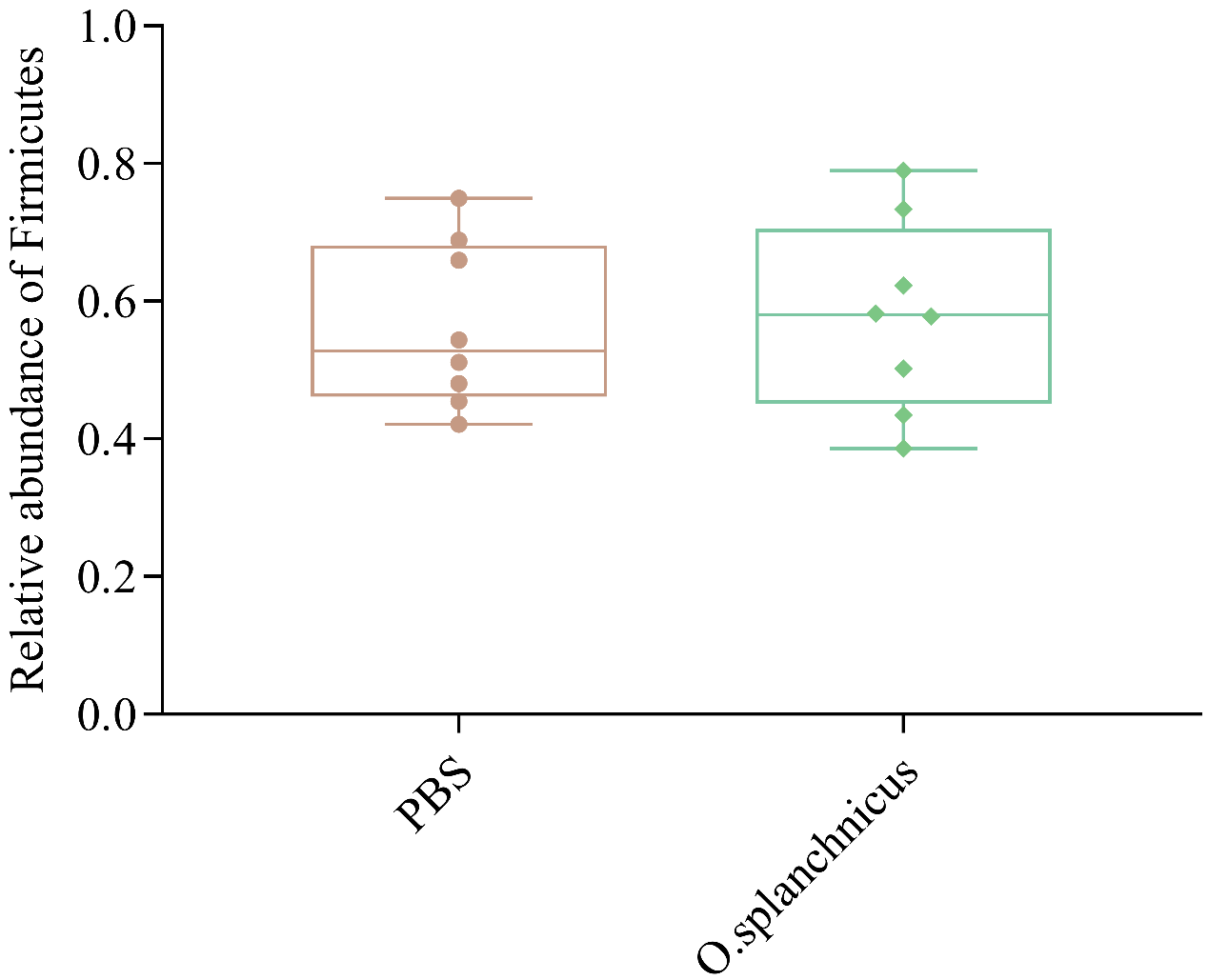


Fig.S11H


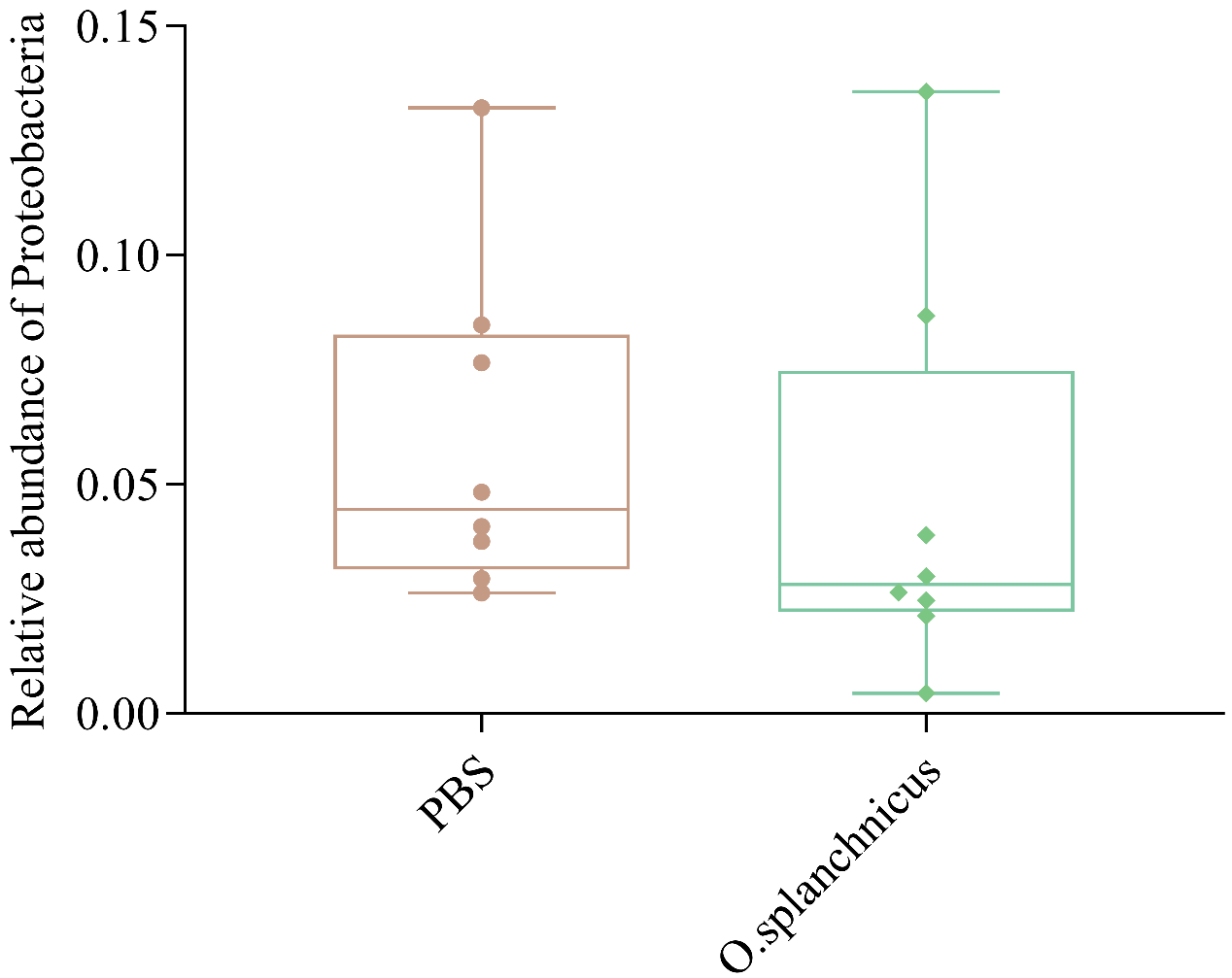


Fig.S11I


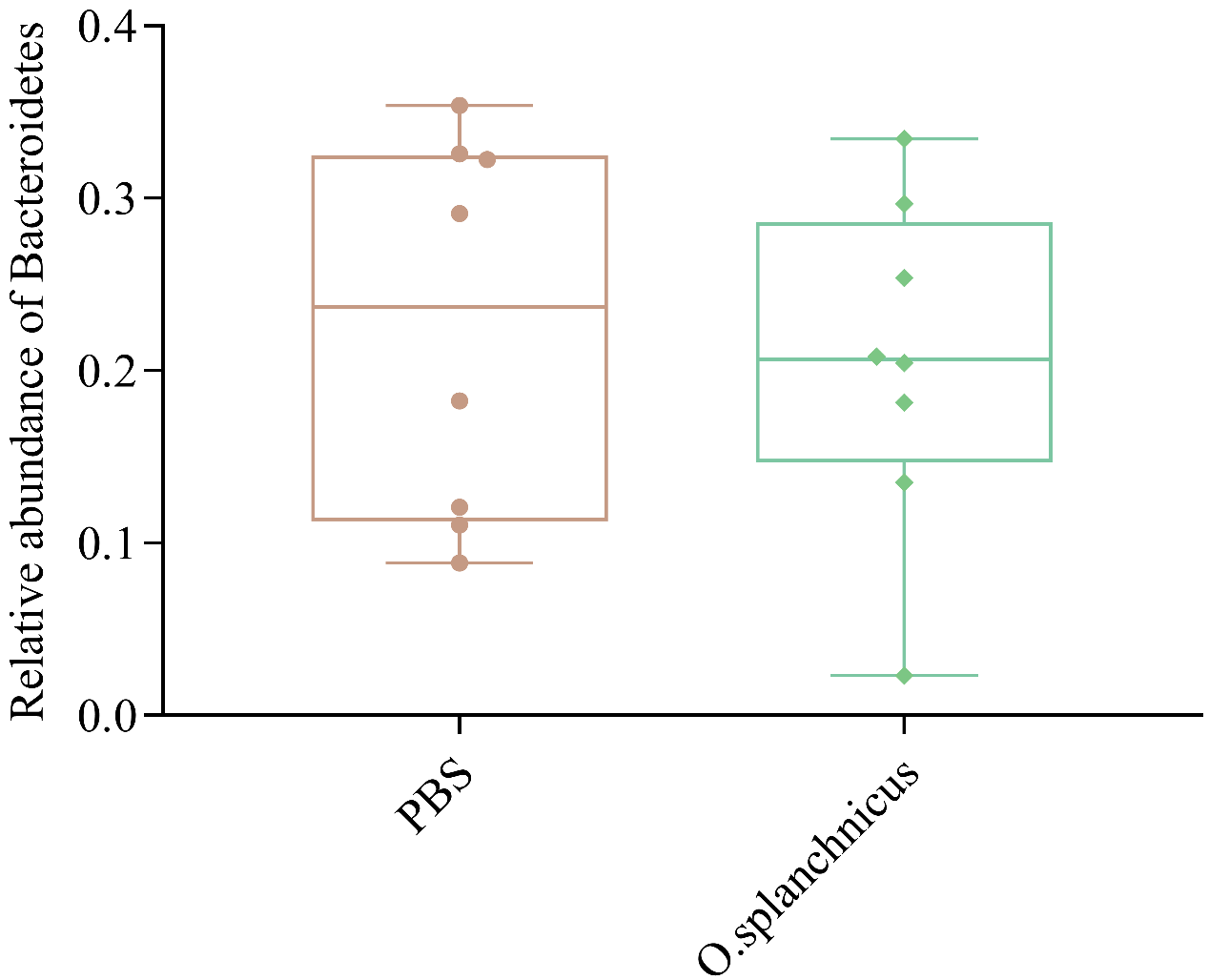


Fig.S12


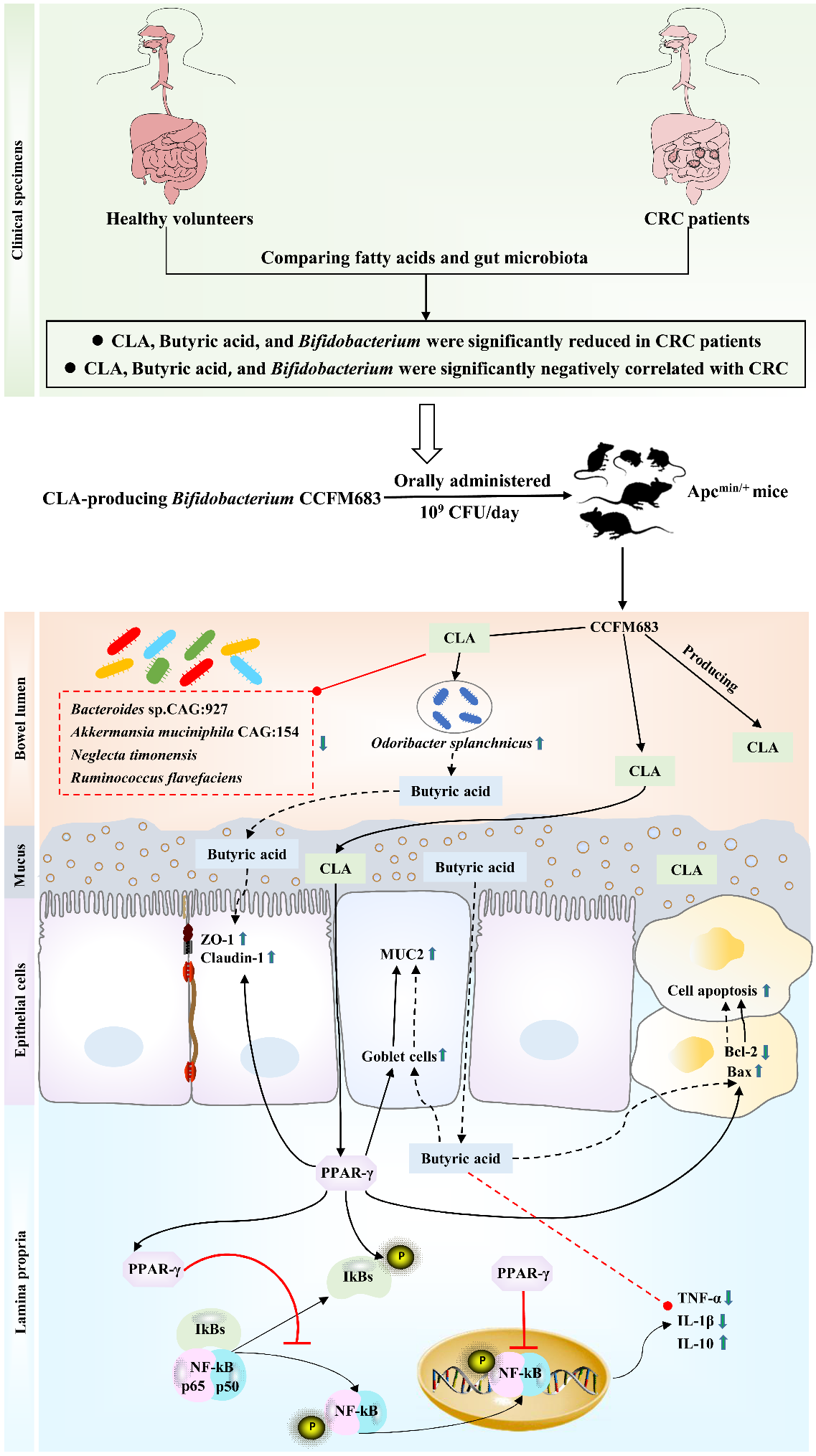

Supplement: Supplemental Material [file KGMI_A_2464945_SM6624.zip › supp s8-s12.docx]

Fig.S4A


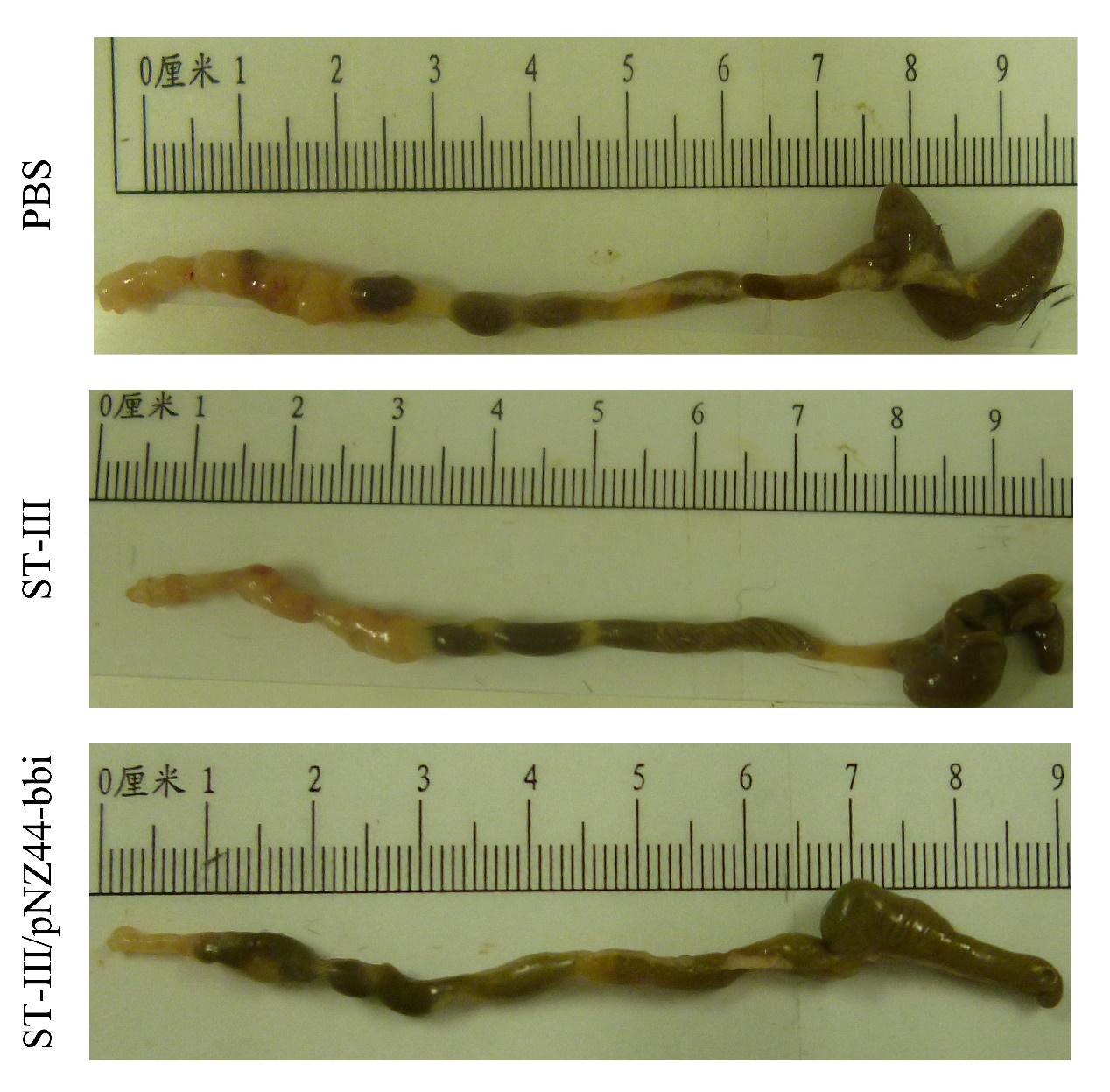


Fig.S4B


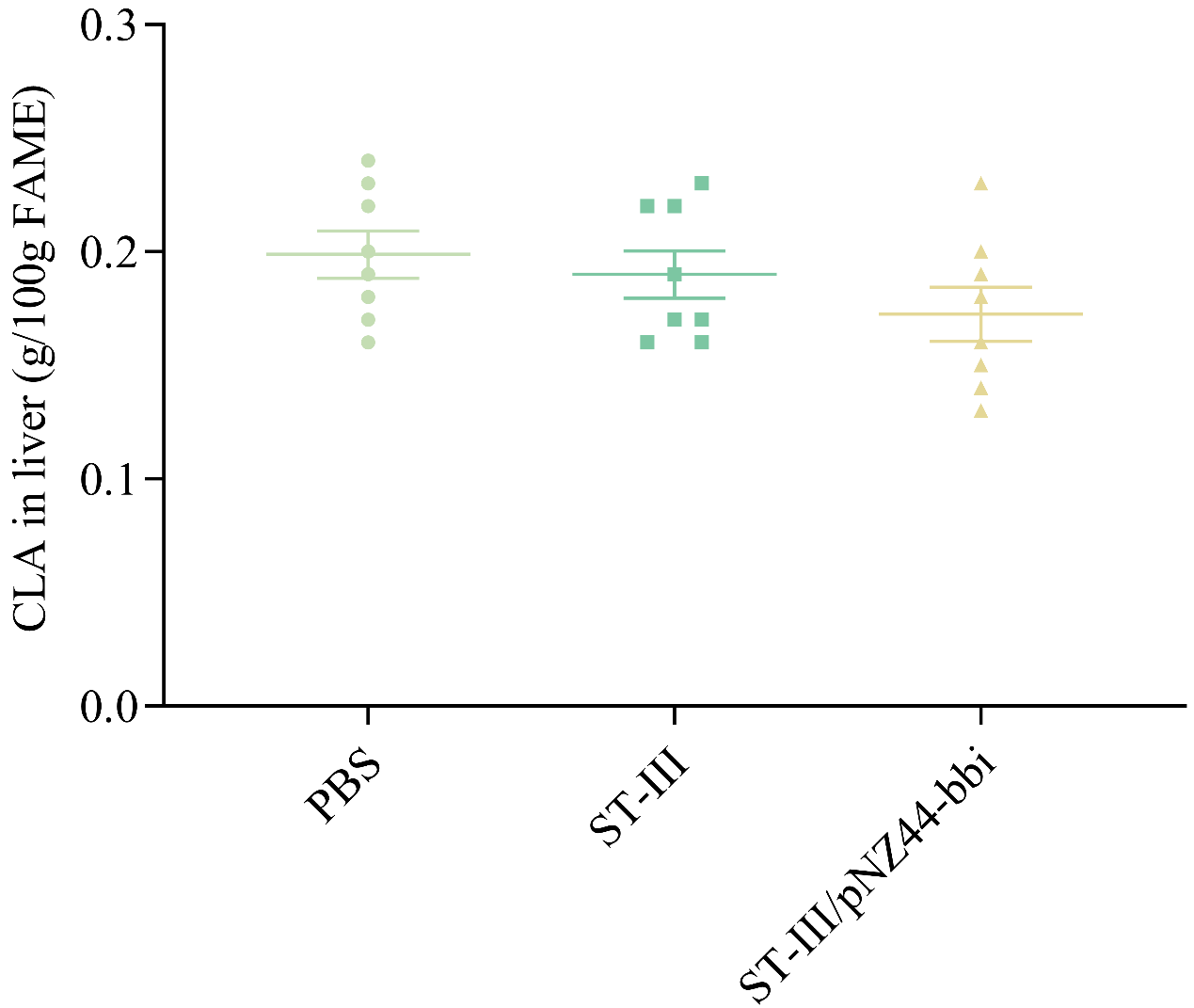


Fig.S4C


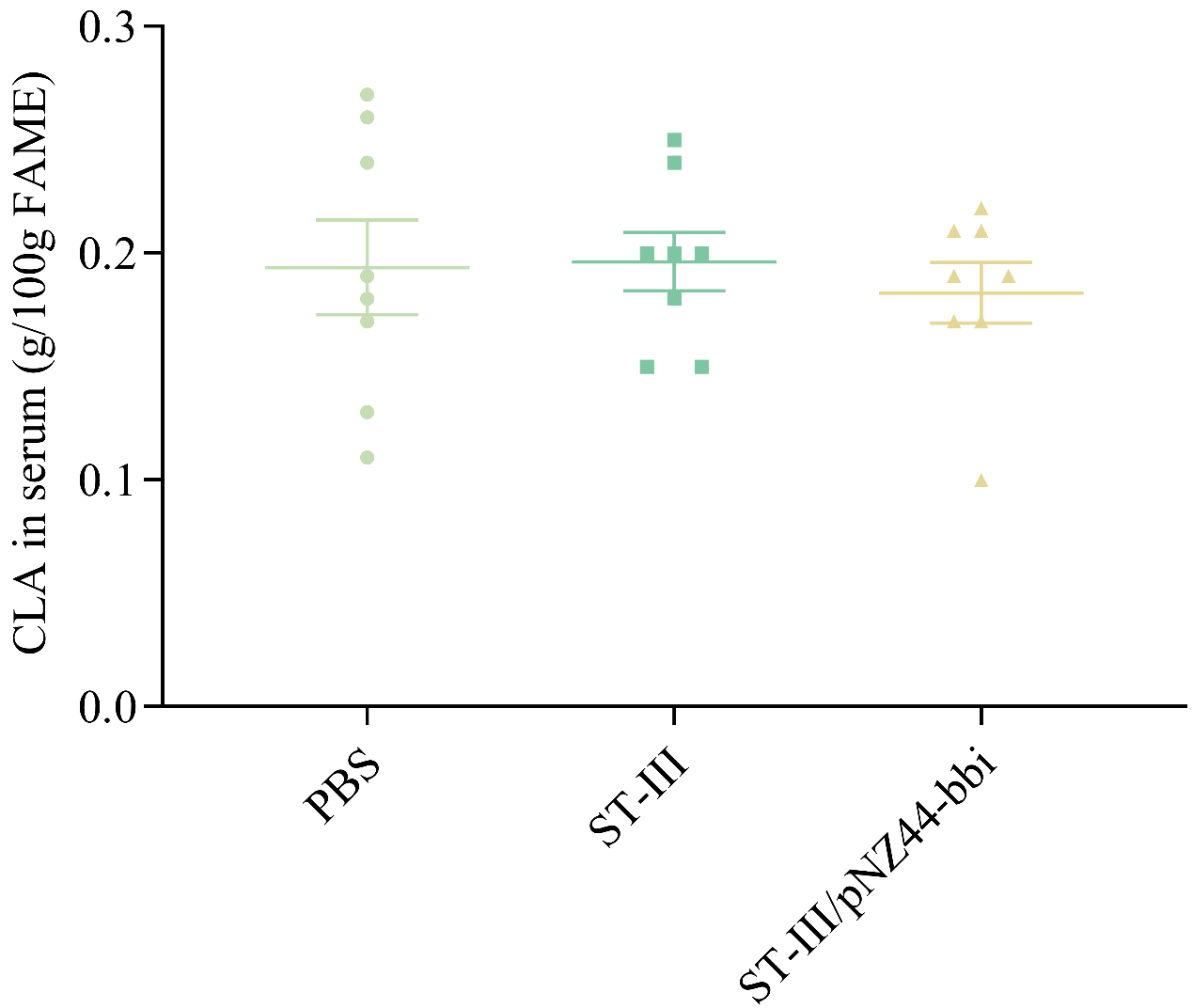


Fig.S5


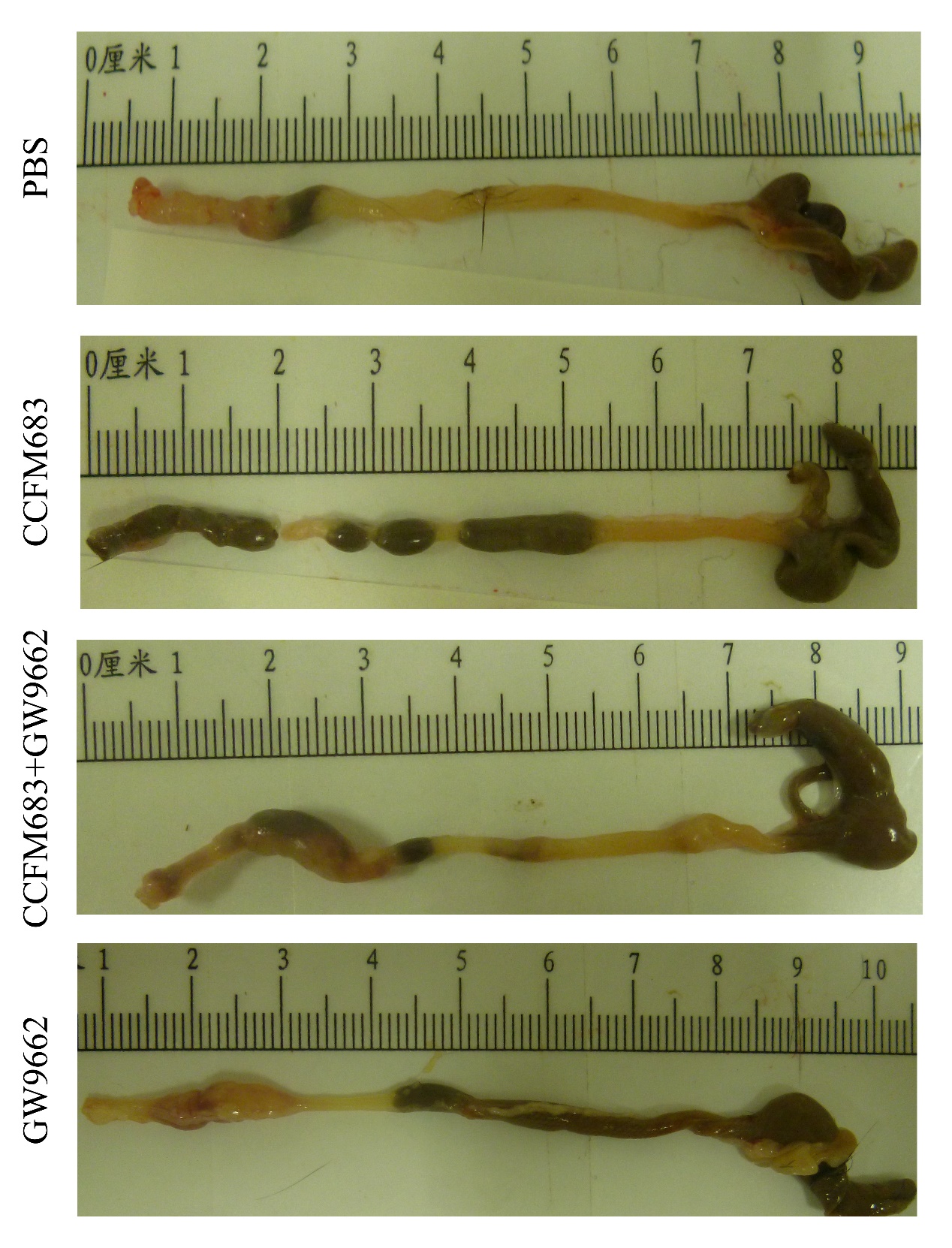


Fig.S6A


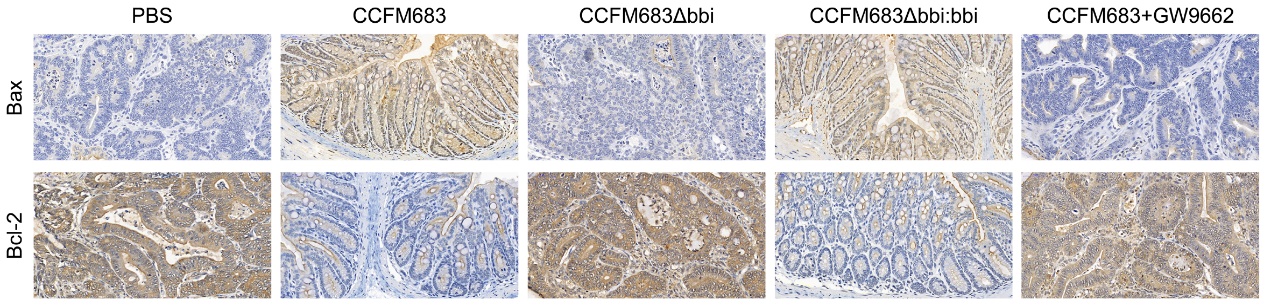


Fig.S6B


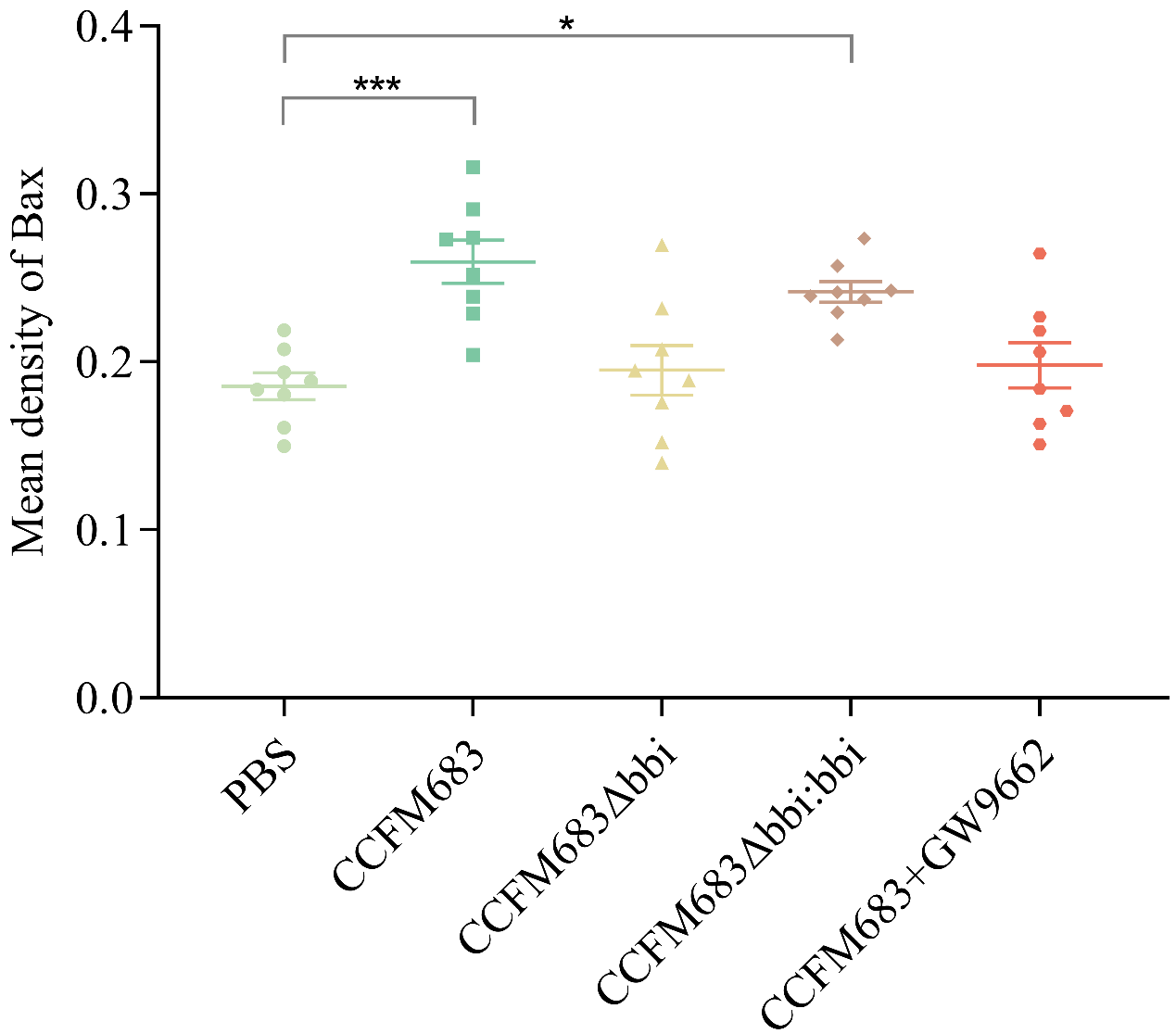


Fig.S6C


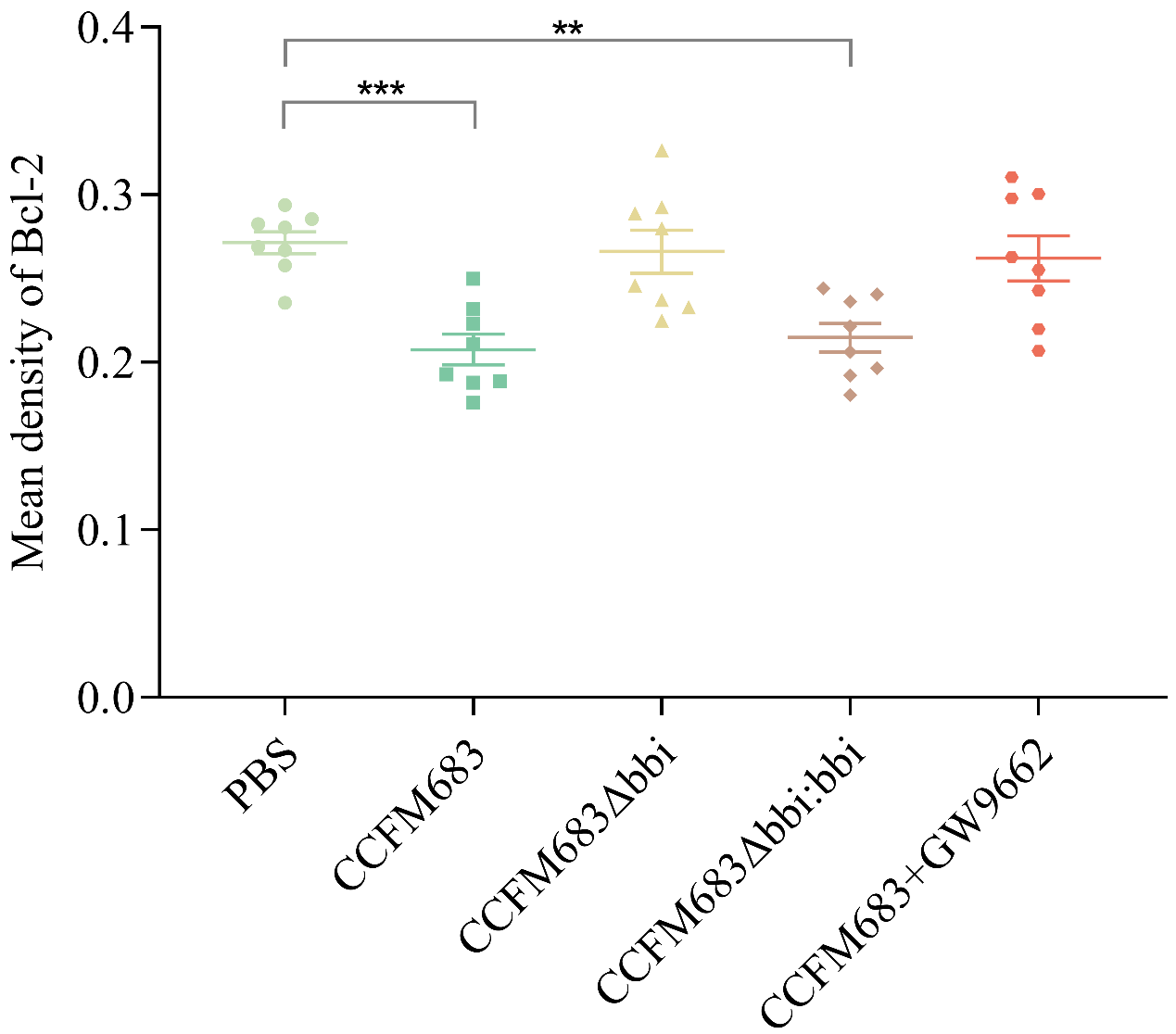


Fig.S7A


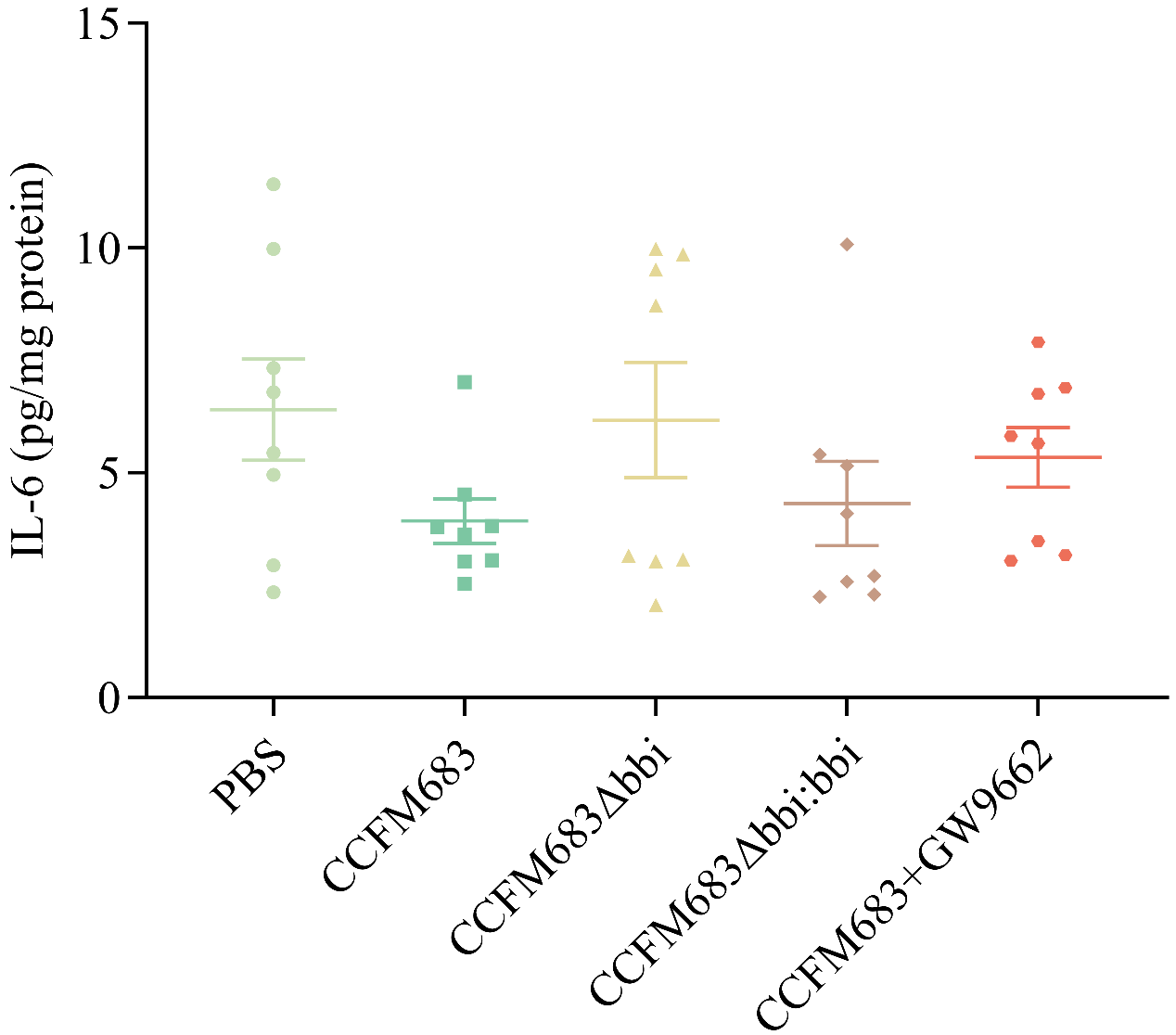


Fig.S7B


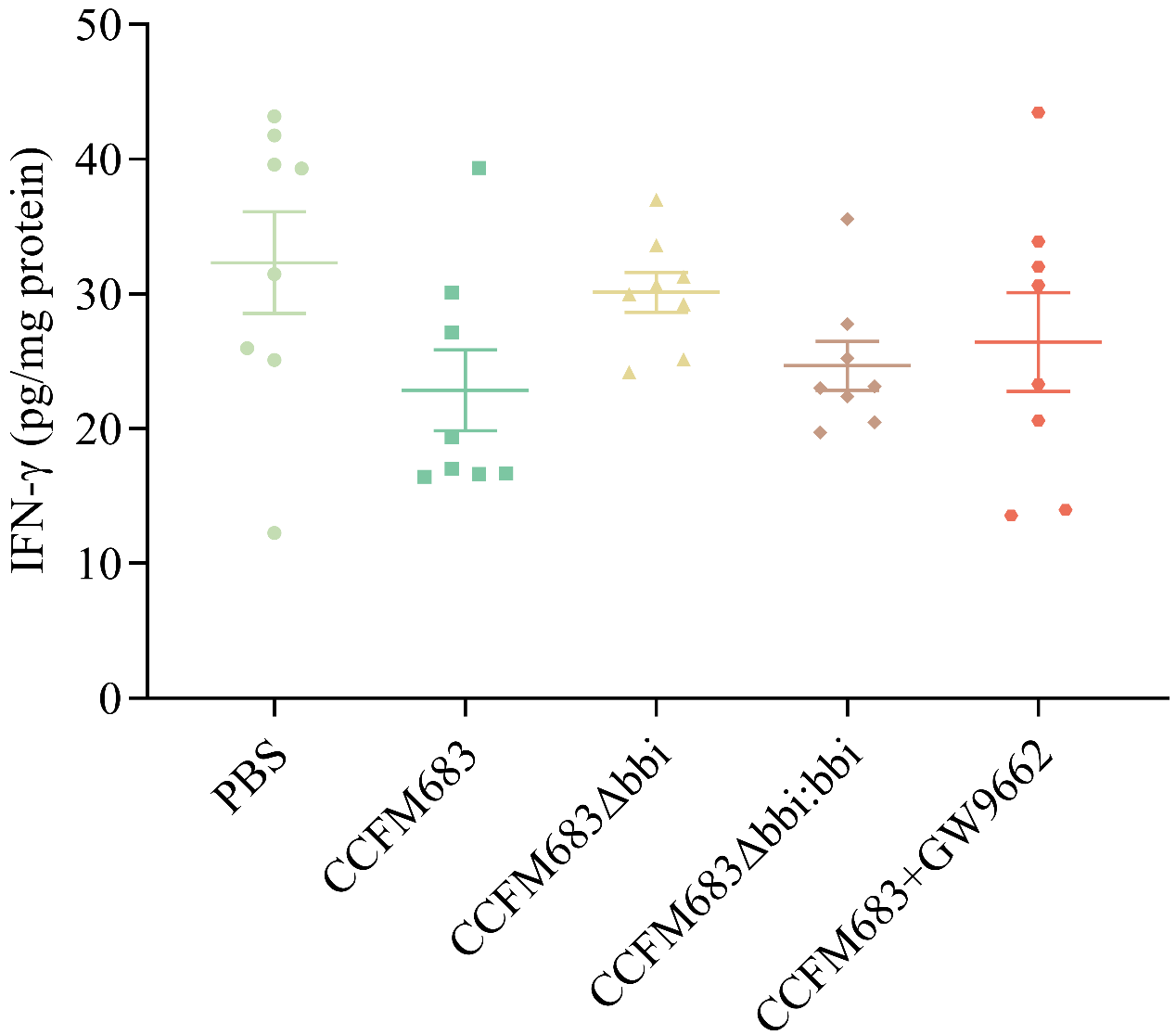

Supplement: Supplemental Material [file KGMI_A_2464945_SM6624.zip › supp s4-s7.docx]
